# Relevance of potential endocytosis motifs in Cedar virus glycoprotein G for its biological activity

**DOI:** 10.1128/jvi.00187-26

**Published:** 2026-04-20

**Authors:** Céline Burrer, Inga Hund, Carolin Rüdiger, Sven Reiche, Martin H. Groschup, Birke A. Tews, Dmitry S. Ushakov, Kerstin Fischer, Sandra Diederich

**Affiliations:** 1 Institute of Novel and Emerging Infectious Diseases, Friedrich-Loeffler-Institut, Federal Research Institute for Animal Health, Greifswald-Insel Riems, Germany; 2 Department of Experimental Animal Facilities and Biorisk Management, Friedrich-Loeffler-Institut, Federal Research Institute for Animal Health, Greifswald-Insel Riems, Germany; 3 Institute of Infectology, Friedrich-Loeffler-Institut, Federal Research Institute for Animal Health, Greifswald-Insel Riems, Germany; 4 Institute of Molecular Virology and Cell Biology, Friedrich-Loeffler-Institut, Federal Research Institute for Animal Health, Greifswald-Insel Riems, Germany; University of Freiburg, Freiburg, Germany

**Keywords:** Cedar virus, glycoprotein, endocytosis, receptor binding, glycoprotein interaction

## Abstract

The glycoprotein (G) and fusion protein (F) of henipaviruses play crucial roles in receptor binding and entry into host cells, also enabling virus spread from cell to cell without the release of infectious particles. For Cedar virus (CedV), the proteolytic activation of the F protein precursor into F1 and F2—and thus, its biological activity—depends on clathrin-mediated endocytosis driven by classical endocytosis motifs YXXΦ and YY in the cytoplasmic tail of the F protein. Similar motifs are present in the cytoplasmic tail of CedV G protein. In this study, we investigated whether these motifs influence CedV G protein expression and transport, endocytosis from the plasma membrane, and overall the biological activity—more specifically receptor binding and mediation of fusion together with the fusion protein. Our data show that the expression of CedV G mutants is comparable to parental G in MDCK cells. Endocytosis can be detected for both parental G and its mutants. However, some G protein mutants show reduced biological activity, as indicated by a decrease in fusion when G mutants are co-expressed with CedV F protein. Interestingly, neither co-expression of CedV F and G mutants on the cell surface nor the binding of G mutants to EFNB2 receptors appears to be compromised. Consequently, the putative endocytosis motifs are not relevant for biological activity of CedV G.

**IMPORTANCE:**

The glycoprotein (G) and fusion protein (F) of henipaviruses mediate host cell entry and direct cell-to-cell virus spread. For the non-pathogenic Cedar virus (CedV), activation of F depends on clathrin-mediated endocytosis mediated by specific YXXΦ and YY motifs in its cytoplasmic tail. The presence of similar motifs in the cytoplasmic domain of CedV G suggested a potential role in G protein trafficking and function. Here, we show that mutation of these motifs does not impair CedV G surface expression, internalization, or receptor binding. However, some mutants showed reduced ability to mediate fusion with CedV F, despite unaltered surface expression and receptor binding. This suggests that these putative endocytosis motifs are not critical for the biological activity of CedV G. These insights will help dissect mechanisms of viral entry and fusion in CedV and contribute to the comparison of these processes with those of the highly pathogenic henipaviruses HeV and NiV.

## INTRODUCTION

*Henipavirus*, a genus within the *Paramyxoviridae* family, includes the highly pathogenic bat-borne Hendra (HeV) and Nipah virus (NiV), two Biosafety Level 4 (BSL4)-classified pathogens with a broad host range and case fatality rates reaching up to 70% in humans (1, 2). Cedar virus (CedV), a closely related henipavirus, was first isolated in 2012 from urine of Australian *Pteropus* bats. Despite its close proximity to HeV and NiV, CedV does not appear to be pathogenic in various animal infection models (3). This characteristic makes CedV a good model virus for its close relatives in research under lower BSL conditions. However, it also raises the question of what underlying molecular determinants are responsible for the difference in pathogenicity. A notable characteristic is the absence of RNA editing capabilities of the phosphoprotein, resulting in a deficiency in V protein coding. This, in turn, leads to an interferon sensitivity of the virus (4). In addition, altered receptor usage could influence virus pathogenicity: while HeV and NiV use the ephrin-B2 (EFNB2) and -B3 (EFNB3) receptors (5–7), CedV can utilize EFNB1, -B2, -A2, and -A5 for cell entry (8). While EFNB1 and -B2 are predominantly expressed in the bladder, lungs, colon, and kidneys, EFNB3 is primarily found in the brain, which aligns with the presence of severe central nervous system pathologies in HeV and NiV infections (9, 10).

A prerequisite for successful infection by henipaviruses is the presence of biologically active glycoproteins in the virus envelope. While the glycoprotein (G) is responsible for binding to the host cell receptor and subsequently triggering refolding of the fusion (F) protein, the F protein leads to fusion of the viral envelope and the host membrane and, ultimately, virus entry. At later stages of the virus life cycle, both proteins lead to cell-to-cell spread without the release of infectious particles, resulting in syncytium formation. For the F protein of several henipaviruses, it has been shown that the inactive precursor F0 needs to be proteolytically activated into F1 and F2 in the endosomal compartment after uptake from the plasma membrane by clathrin-mediated endocytosis (CME) (11–17). CME serves as machinery for sorting proteins from the cell surface to the cytoplasm or other organelles, such as the early endosome or Golgi apparatus, where the proteins are directed to their final destination or modified and packaged for transport. Finally, recycling of proteins to the cell surface is enabled by the late endosome (18–21). The onset of endocytosis is based on the recognition of specific motifs (22). These signals can be tyrosine-based, such as YXXΦ (where Y stands for tyrosine, X for any amino acid, and Φ for a hydrophobic amino acid), or dileucine-based (LL), and are recognized by adaptor protein (AP) complexes (AP-1 and AP-2) or Golgi-localized, γ-ear-containing, Arf (ADP-ribosylation factor)-binding proteins (GGAs), respectively (22, 23). In particular, the tyrosine-based YXXΦ-motifs within the cytoplasmic domains of HeV F, NiV F, and CedV F appear to play a significant role for their endocytic uptake and, furthermore, for their expression on the cell surface and biological activity (11, 12, 24). More precisely for CedV F, it has been shown that in addition to the YXXΦ-motif, a di-tyrosine motif (YY) also seems to have a strong effect on endocytosis and, thus, biological activity, which is drastically reduced in the absence of these motifs (24). The glycoprotein (G) of henipaviruses, a tetrameric structured protein, binds in its metastable form to the host cell receptor, triggering the F protein, which then leads to membrane fusion (25). Consistent with the observations for CedV F, CedV G also contains potential endocytosis motifs in its cytoplasmic domain including a YXXN motif (Y_9_XXN), which is a degenerate form of the typical YXXΦ motif, a di-tyrosine motif (Y_47_Y_48_), and a di-leucine motif (L_59_L_60_). While the YXXN motif has been shown to have negligible effects on endocytosis in CedV F (24), it has been described as an important endocytosis signal in the measles virus hemagglutinin (MeV H) (26). In the case of the human immunodeficiency virus type 1 (HIV-1), a di-leucine motif appears to play a critical role for endocytosis (27), whereas it does not appear to be important for this mechanism for NiV G (11).

In this study, we investigated the role of the three potential endocytosis motifs for endocytosis, receptor binding, and the biological activity of CedV G. The latter is defined by the formation of multinucleated giant cells, so-called syncytia, which only arise when functional receptor binding and triggering of the CedV F protein into its fusion state occur. To this end, plasmids encoding for CedV G mutants with disrupted motifs in the cytoplasmic domain, either individually or in all possible combinations, were generated and proteins analyzed. Our results show no effect of the missing motifs on expression or internalization of the proteins. However, the mutants lacking the di-tyrosine motifs (Y_47_Y_48_) seem to be impaired in syncytium formation and thus in biological activity.

## RESULTS

**Fig 1:**
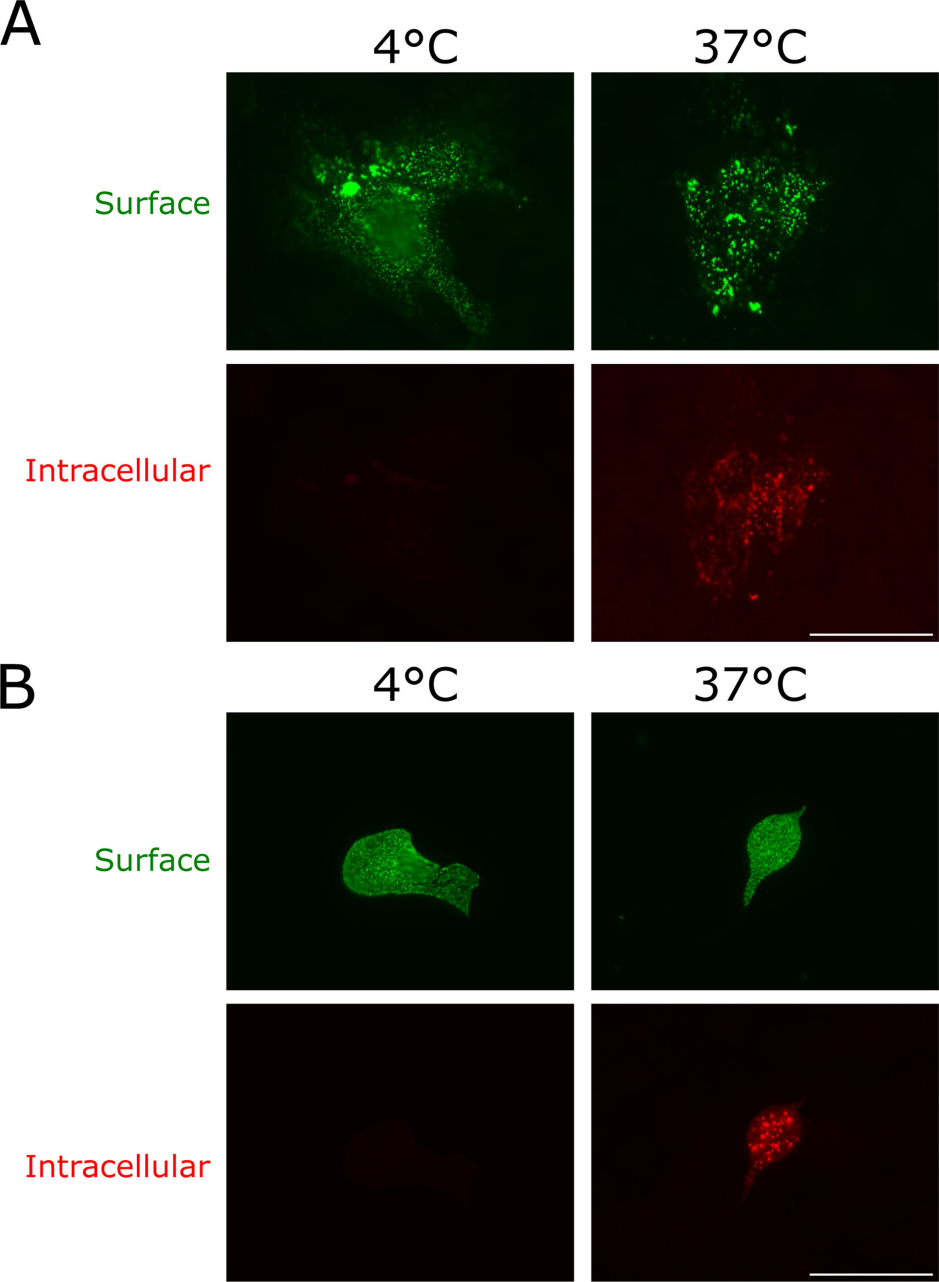
Endocytosis of CedV G protein in infected (**A**) or transfected (**B**) MDCK-2 cells. (**A**) At 24 h p.i., rCedV-infected cells were incubated with CedV G-specific monoclonal antibodies to label G protein on the cell surface. Then, cells were either incubated at 4°C or shifted to 37°C for 30 min to allow endocytosis to occur. Primary antibodies bound to G protein on the cell surface were stained with AlexaFluor (AF) 488-conjugated secondary antibodies. After fixation and permeabilization, internalized primary antibody-CedV G protein complexes were visualized with AF 568-conjugated secondary antibodies. Magnification 60×. Scale bar = 50 µm. *n* = 1. (**B**) At 24 h p.t., cells were incubated with an anti-HA tag antibody to label G protein on the cell surface, and the following protocol was performed as described for panel A. Magnification 60×. Scale bar = 50 µm. *n* = 3.

It has been shown that endocytosis of CedV F plays a crucial role in its biological activity and, consequently, in the infectivity of the virus. To analyze if the other glycoprotein, CedV G, also undergoes endocytosis, we performed a qualitative antibody uptake assay. MDCK-2 cells were either infected with recombinant CedV or transfected with CedV G encoding plasmid DNA and incubated for 24 h. Then, G proteins expressed on the cell surface were stained with anti-CedV G-specific monoclonal primary antibodies (for infection) or with an anti-HA tag primary antibody (for transfection). In the next step, endocytosis was allowed to occur for 30 min by shifting the cells to 37°C. Primary antibodies on the cell surface were incubated with AF 488-conjugated secondary antibodies in surplus. After cell fixation and permeabilization, internalized G protein–primary antibody complexes were visualized with AF 568-conjugated secondary antibodies. Cells incubated at 37°C showed both green fluorescent signals for surface protein and red fluorescent staining of intracellular particles in both the infection (Fig. 1A) and the transfection assays (Fig. 1B). In contrast, cells kept at 4°C only show green cell surface staining in both assays. This indicates endocytosis of the CedV G protein. To exclude an influence of the HA tags on the internalization behavior of the transiently expressed G protein, this assay was also conducted with untagged CedV G proteins. The results shown in Fig. S1 demonstrate that there is no difference in endocytosis when comparing tagged and non-tagged G proteins.

**Fig 2:**
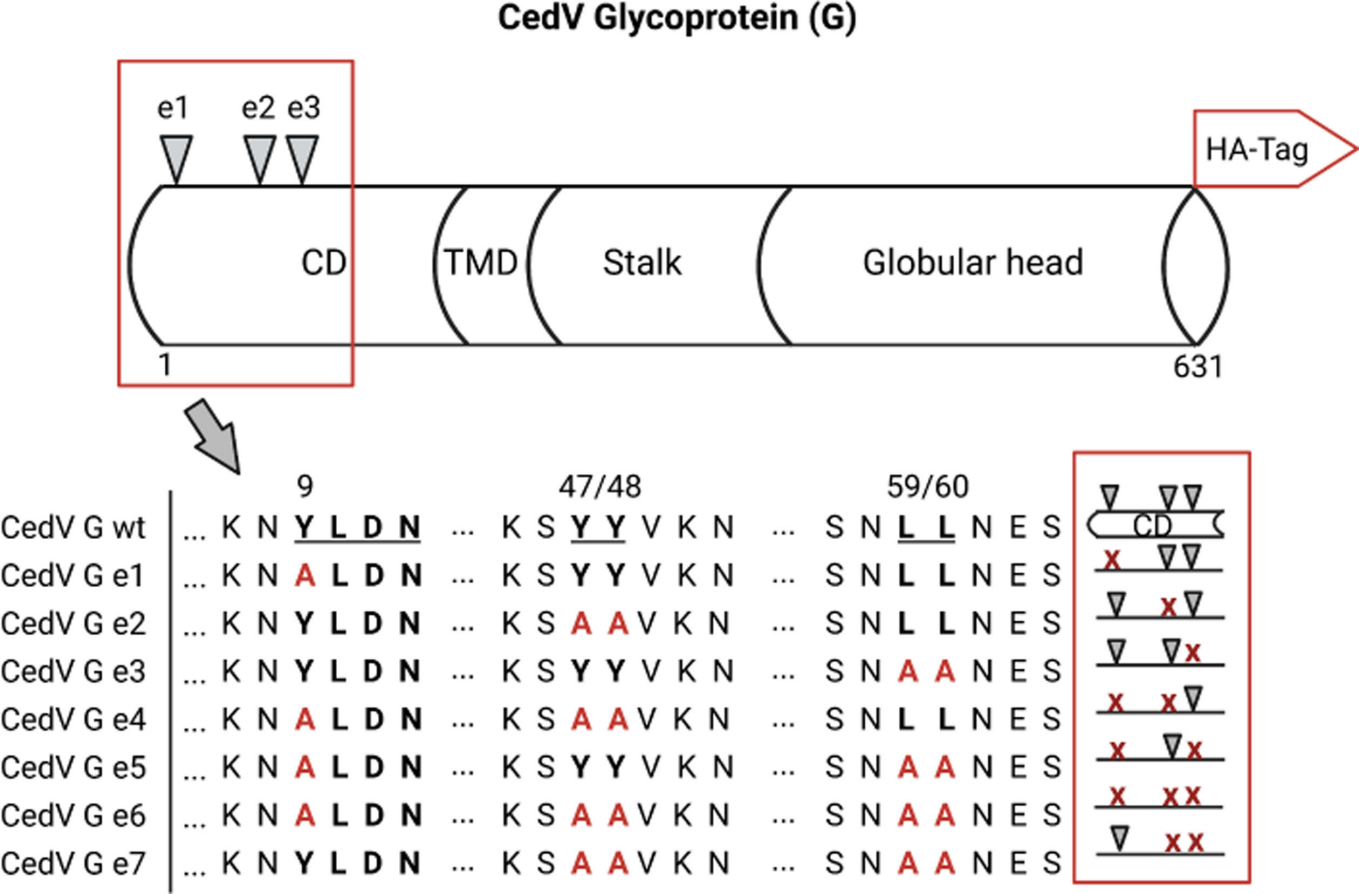
Schematic overview of the CedV G endocytosis motif mutants generated in this study. Putative tyrosine- and leucine-based motifs are underlined. All mutants include a C-terminal hemagglutinin (HA) tag for detection. CD, cytoplasmic domain; TMD, transmembrane domain; wt: wild-type. Created in BioRender. Burrer, C. (2026) https://BioRender.com/cr75tii.

To see if the tyrosine- and leucine-based motifs within the CedV G protein cytoplasmic tail are required for endocytosis, seven G protein mutants were generated by replacing tyrosine or leucine in the motifs with alanine (Fig. 2). Mutants displayed either single (e1, e2, and e3) or multiple (e4, e5, and e7) mutations, as well as a mutant lacking all putative endocytosis signals (e6), to investigate the potential additive effects of these mutated motifs (Fig. 2). Apart from a degenerate form of typical YXXΦ motif, a YXXN motif (Y_9_XXN), which was described to affect endocytosis of the measles virus hemagglutinin (26), we also chose to substitute the di-tyrosine residues at amino acid positions 47–48 (Y_47_Y_48_), which we recently demonstrated to be important for CedV F uptake and biological activity (24). In addition, we mutated the di-leucine residues at positions 59–60 (L_59_L_60_), as these motifs are well-known sorting signals for several cellular mechanisms (22) and are also critical for endocytosis of the human immunodeficiency virus type 1 (HIV-1) envelope protein (27).

**Fig 3:**
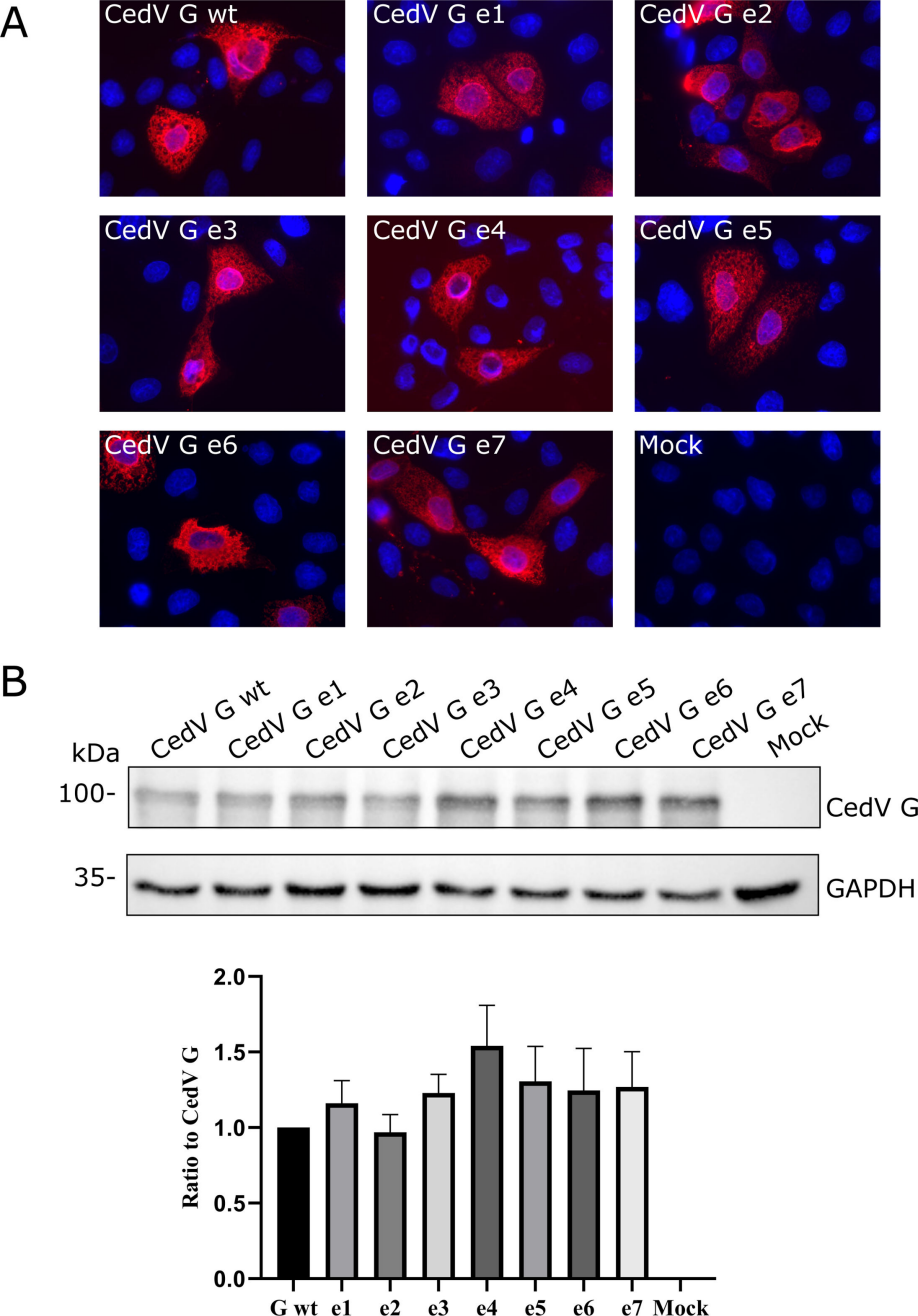
Expression of CedV G mutants. (**A**) Indirect immunofluorescence analysis of CedV G and mutant CedV G proteins. At 24 h p.t., MDCK-2 cells expressing different CedV G proteins were fixed and stained with anti-HA tag-specific antibodies and with AF568-conjugated secondary antibodies. Nuclei were stained with DAPI. Magnification 60×. Scale bar = 50 µm. *n* = 2. (**B**) Western blot analysis of lysates from CedV G mutant proteins expressed in MDCK-2 cells. After separation of the proteins by 10% SDS-PAGE under reducing conditions, proteins were transferred to nitrocellulose and detected with an anti-HA tag antibody and HRP-conjugated secondary antibody. Proteins were visualized with anti-HA tag-specific primary, HRP-labeled secondary antibodies, and chemiluminescence. The expression was normalized to the GAPDH loading control (anti-GAPDH antibody, 1:5,000) and quantified in comparison to CedV G. *n* = 6.

To assess the overall expression of CedV G and G mutants, an indirect immunofluorescence in MDCK-2 cells was carried out at 24 h post-transfection (p.t.). Red fluorescence specific to the CedV G proteins could be detected to the same extent in all samples (Fig. 3A). Furthermore, to quantify potential differences in expression levels of the different mutants compared to parental CedV G, lysates of transfected MDCK-2 cells were analyzed in Western blot. CedV G bands were normalized to GAPDH, which served as loading control, and then compared to the parental CedV G, which was set to 1 (Fig. 3B). In accordance with the findings of the immunofluorescence, bands with a molecular weight of 100 kDa corresponding to the expected molecular weight of CedV G were identified for CedV G wt and all G mutants. Quantification of the expression levels only showed negligible, statistically non-significant differences (adjusted *P* value > 1 for all mutants compared to CedV G wt) between mutants and parental CedV G (Fig. 3C).

**Fig 4:**
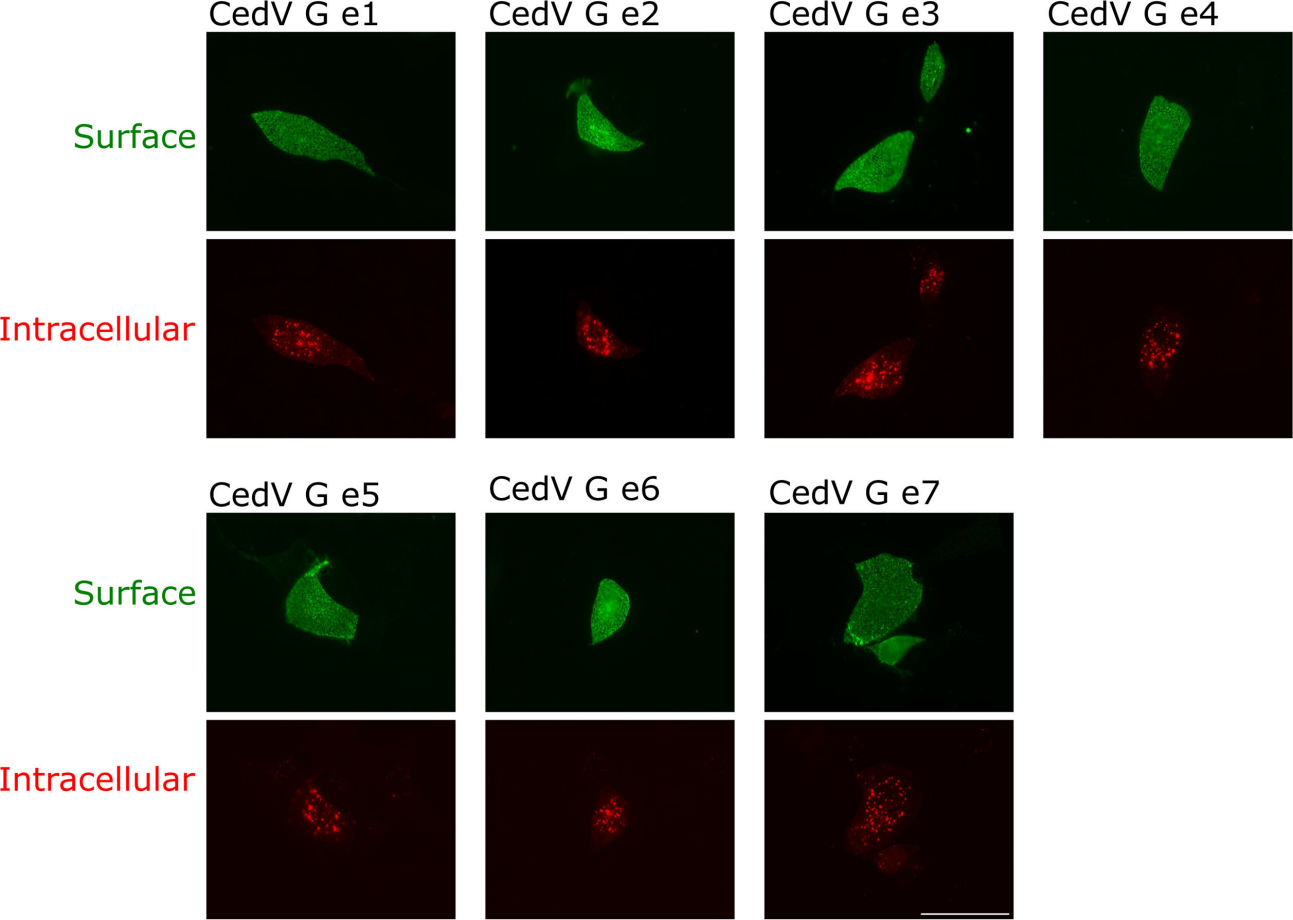
Endocytosis assay of CedV G mutants in MDCK-2 cells. At 24 h p.t., G-expressing cells were incubated without prior fixation with an anti-HA tag antibody. Then, cells were either incubated at 4°C or shifted to 37°C for 30 min to allow endocytosis to occur. Bound antibodies on the cell surface were stained with AF 488-conjugated secondary antibodies, while endocytosed proteins-antibody complexes were visualized with AF 568-conjugated secondary antibodies after cell fixation and permeabilization. Magnification 60×. Scale bar = 50 µm. *n* = 3.

The effect of individual and multiple mutations in the cytoplasmic domain of CedV G protein on endocytosis was studied in another qualitative antibody uptake assay, as described above, using all generated mutants. For all mutants, green fluorescence on the cell surface was detected. After a 30-min incubation period at 37°C, red fluorescent intracellular vesicles were also observed for all mutants (Fig. 4). Therefore, disruption of any of the motifs (Y_9_XXN, Y_47_Y_48_, and L_59_L_60_), either individually or in combination, had no effect on internalization of the mutants. To evaluate whether co-expression of CedV F protein affects uptake of G or G mutants, we performed another antibody uptake assay with cells co-expressing F and G proteins (Fig. S2). Green fluorescence on the cell surface, as well as red fluorescence representing internalized G proteins, was detected in the samples incubated at 37°C, indicating that the co-expression of CedV F had no effect on uptake of CedV G (mutant) measured qualitatively.

**Fig 5:**
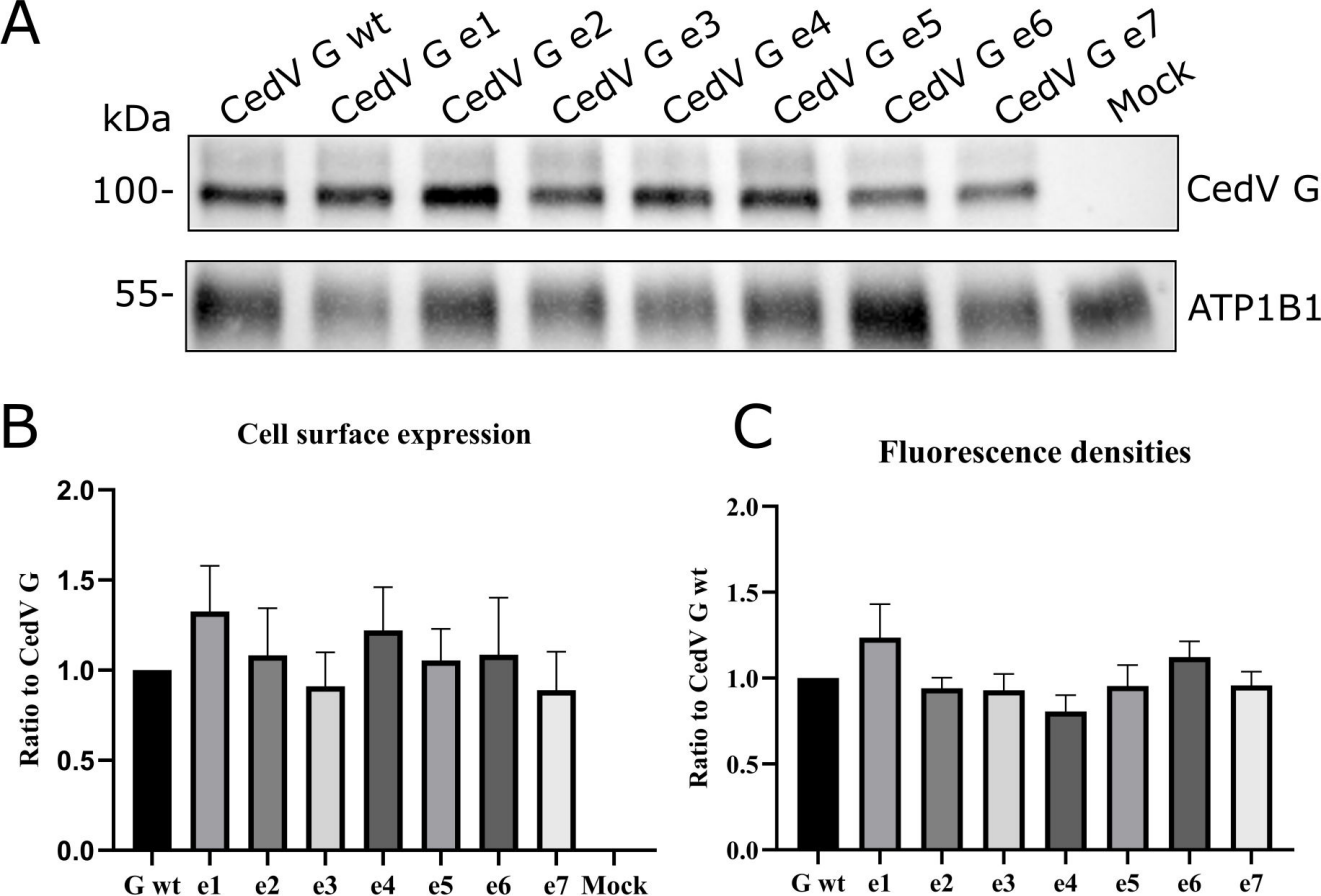
Cell surface expression of CedV G proteins. (**A**) At 30 h p.t., MDCK-2 cells expressing G proteins were labeled with biotin on ice. After lysis, biotinylated proteins were immunoprecipitated using NeutrAvidin beads. After separation on 10% SDS page under reducing conditions, G proteins were visualized in a Western blot with anti-HA tag-specific primary, HRP-labeled secondary antibodies, and chemiluminescence. ATP1B1 (1:5,000) is used as loading control. (**B**) For quantification, protein levels were normalized to the loading control and parental CedV G was set to 1. A representative blot is shown from *n* = 3 independent experiments. (**C**) Additional quantification through imaging of cell surface staining in IFA was performed using the 4°C cell controls from Fig. 4. Mean fluorescence densities of five representative fields of view from each mutant were evaluated and compared to parental CedV G, which was set to 1. *n* = 1.

Transport of the glycoprotein G to the cell surface is indispensable for its functionality, receptor binding, and thus the biological activity of the protein. Cell surface levels of CedV G and G mutants were evaluated 30 h after transfection by performing a cell surface biotinylation assay. Biotinylated proteins were immunoprecipitated from cell lysates using NeutrAvidin beads and then analyzed in a Western blot using a primary antibody against the HA tag for detection. The sodium/potassium-transporting ATPase subunit beta 1 (ATP1B1) served as loading control and was used for normalization of the mutant CedV G protein expression. Adjusted volumes of the mutant proteins were compared to the parental CedV G protein expression level, which was set to 1 (Fig. 5). As expected from the antibody-uptake assay, we were able to detect G and all G mutants at the cell surface (Fig. 5A). Moreover, the level of expression of the G mutants only exhibited minimal, statistically non-significant variations (adjusted *P* value > 1 for all mutants compared to CedV G wt) in comparison to that of the parental G protein, indicating that the motifs in the cytoplasmic domain do not interfere with cell surface transport in MDCK-2 cells (Fig. 5B).

To measure differences in surface expression in a different approach, the protein located on the cell surface was additionally quantified by cell surface immunofluorescence staining. The 4°C control cells from the qualitative endocytosis assay (no endocytosis was allowed) were used, and the averaged fluorescence densities of CedV G mutant proteins were compared to parental CedV G protein surface expression, which was set to 1 (Fig. 5C; Fig. S3). Similar to the results of the parental CedV G protein, all mutants were at the same levels on the cell surface with only marginal differences.

**Fig 6:**
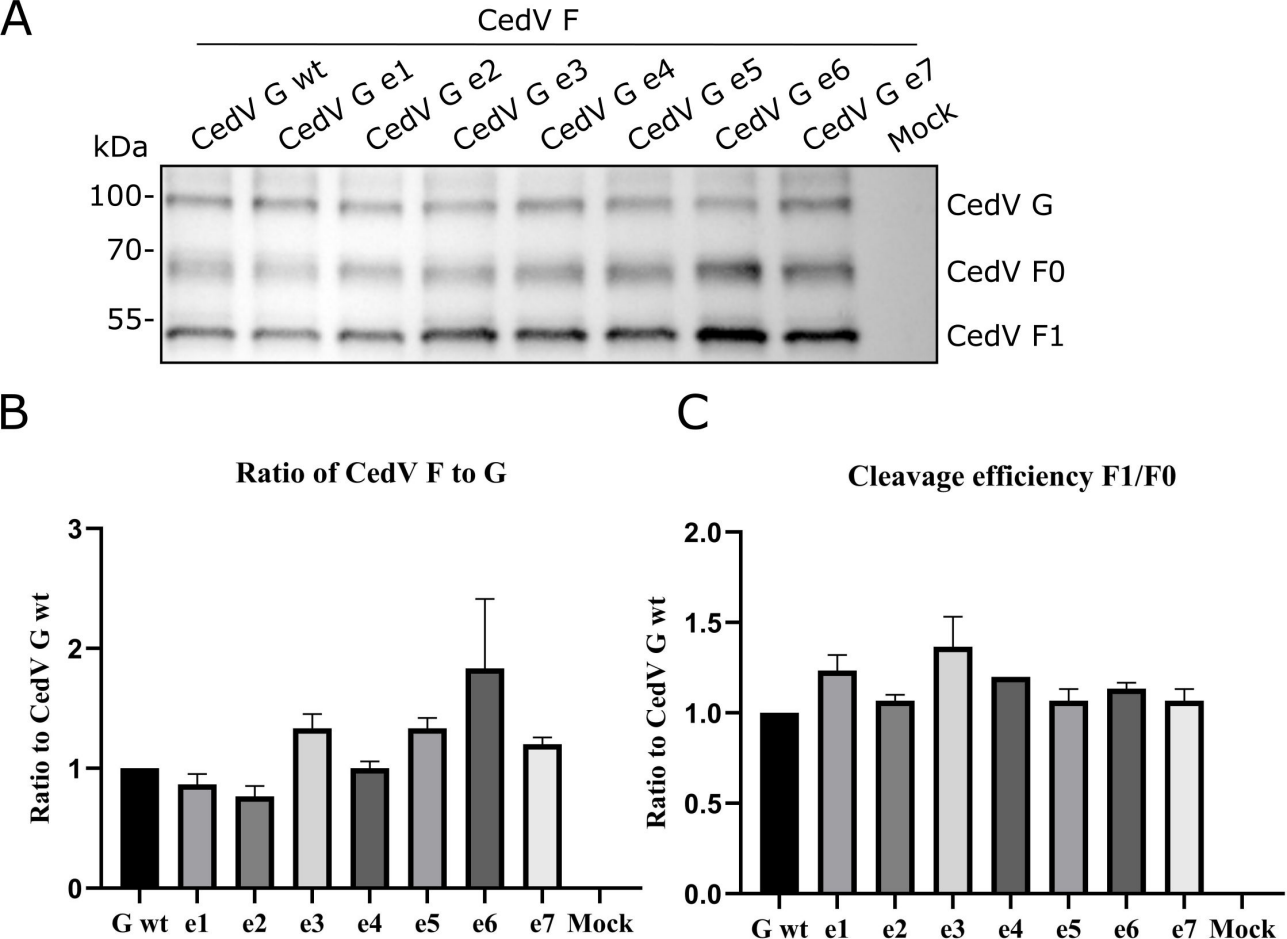
Co-expression of CedV F and G proteins at the cell surface of MDCK-2. At 30 h p.t., cells were labeled on ice with biotin. After lysis, biotinylated proteins were immunoprecipitated using NeutrAvidin beads and analyzed in SDS-PAGE under reducing conditions. (**A**) CedV G and F proteins were visualized with anti-HA tag-specific primary, and HRP-labeled secondary antibodies. (**B**) Total F protein levels were normalized to the respective G mutant. Parental CedV G was set to 1. (**C**) To assess the effect on F protein processing, CedV F1 to F0 ratios were determined and compared individually to parental CedV G. *n* = 3.

The presence of both glycoproteins, G and the fusion protein F, is a prerequisite for CedV to bind to and enter host cells and, at later stages of virus replication, for virus spread from cell to cell, leading to syncytium formation. Consequently, it is of interest to assess the cell surface expression of the F protein when co-expressed with the G mutants. For this, a surface biotinylation assay in MDCK-2 cells co-expressing CedV F and CedV G or mutant CedV G proteins was performed at 30 h p.t. Protein lysates were analyzed under reducing conditions by SDS-PAGE and Western blot. As shown in Fig. 6A, CedV G and G mutants were detectable on the cell surface in comparable amounts as also seen in Fig. 5A. Further, CedV F0 and the cleavage product F1 can be detected on the cell surface independently if co-expressed with CedV G or G mutants. In order to assess the influence of the different endocytosis motifs in the cytoplasmic domain of G on the surface expression level of the fusion protein, total F protein was set in ratio to the expression level of the respective G protein (Fig. 6B). Despite the presence of minimal non-significant variations (adjusted *P* values > 1), expression levels of CedV F co-expressed with all CedV G mutants are comparable to F proteins co-expressed with CedV G wt. To achieve an estimate of F processing, CedV F1 was set in relation to F0 and then compared to the expression ratios of CedV F1 to F0 co-expressed with parental G protein (Fig. 6C). Overall, analysis of F processing in the presence of CedV G mutants revealed only minor, non-significant variation (adjusted *P* values > 1). Thus, we conclude that endocytosis motifs in the cytoplasmic domain of G have no impact on F transport to the cell surface or F processing, which requires transport through the early endosomes, nor does F co-expression influence G or mutant G surface expression. In addition, the overall expression levels of CedV G and mutant CedV G proteins at the cell surface were assessed by quantification from cell surface staining of MDCK-2 cells co-expressing CedV F and G or G mutants (4°C controls from qualitative endocytosis assay), as described above. As presented in Fig. S4A, all mutants show comparable surface expression levels when set in relation to CedV G wt levels. A selection of representative images used for quantification is presented in Fig. S4B.

**Fig 7:**
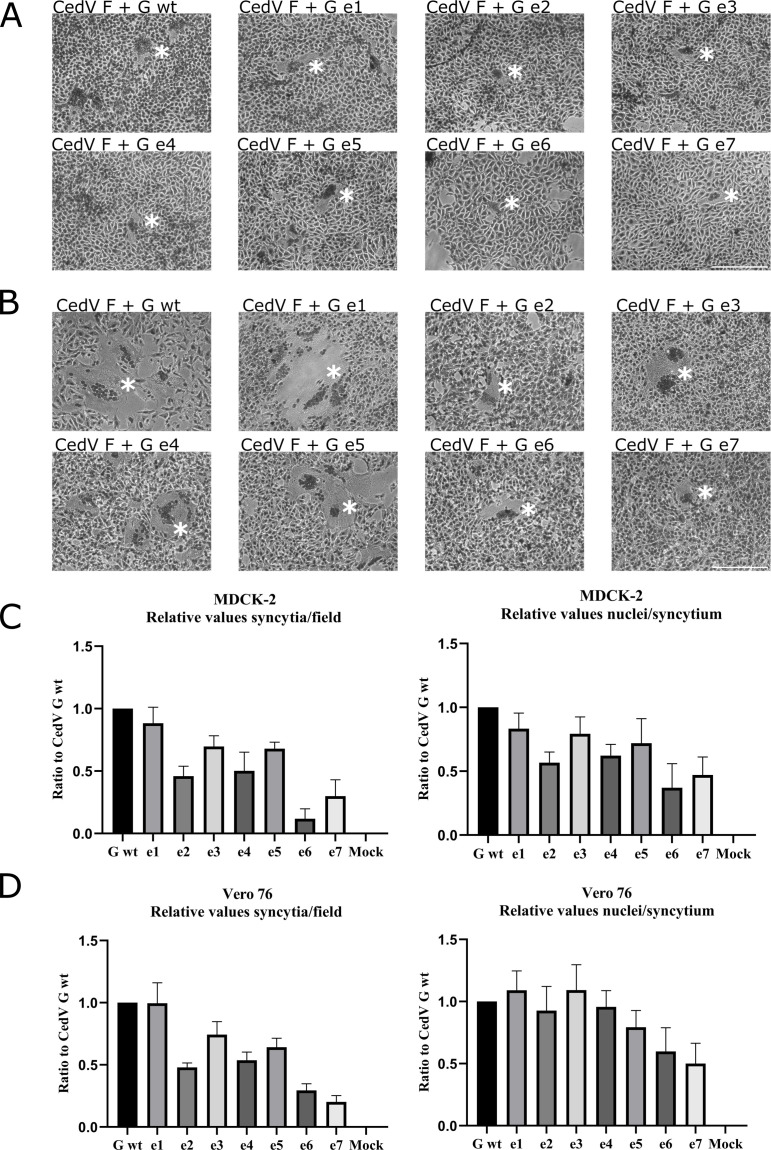
Fusion activity of CedV G and G mutants. Syncytium formation in MDCK-2 (**A**) and Vero 76 cells (**B**) co-expressing CedV F and CedV G or G mutants was visualized by Giemsa staining at 48 h p.t. * indicates an example of a syncytium in each image. Magnification 20×. Scale bar = 200 µm. *n* = 5. Quantification of syncytium formation in MDCK-2 (**C**) and Vero 76 cells (**D**) co-expressing CedV F and (mutant) CedV G: In five representative vision fields for each mutant, the number of syncytia and the number of cell nuclei per syncytium were counted at a 20× magnification, and values from five biological replicates were averaged. The mean value of the parental CedV G co-transfected with CedV F was set to 1, and the mutant values were set in relation to this value. All *P* values >0.05 by Mann-Whitney *U* test after Bonferroni correction.

**Fig 8:**
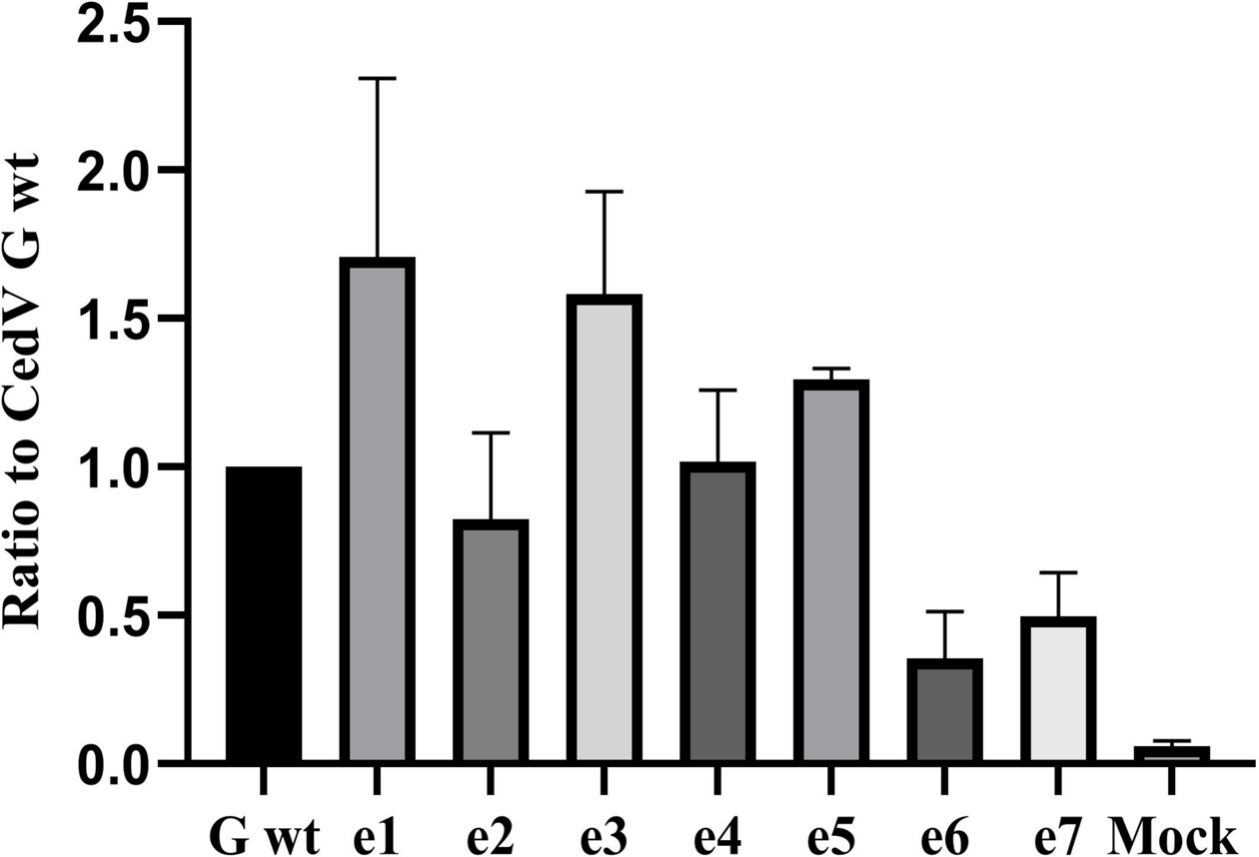
Quantitative, luciferase reporter gene-based fusion assay of CedV G and G mutants. Vero 76 cells were co-transfected with plasmids (pCite Renilla) encoding for Renilla luciferase gene under control of a T7 promoter, CedV F, and CedV G or G mutants. After 48 h, T7-expressing cells were layered onto glycoprotein-expressing cells and incubated for 3 h to allow for cell fusion. After cell lysis, luciferase activity was measured in a luminometer. Samples were tested in duplicates in three independent experiments. Luciferase activity measured for the parental CedV G protein co-transfected with CedV F protein was set to 1 and served as a reference for fusion activity in comparison to the mutants. Values include the standard error of the mean (SEM). All *P* values > 0.05 by Mann-Whitney *U* test after Bonferroni correction. Mock: Background activity assessed with cells transfected with pCAGGS CedV G and pCite Renilla only, layered with T7 polymerase expressing cells. *n* = 3.

To further evaluate if the absence of particular endocytosis motifs in the cytoplasmic domain of the receptor-binding protein G has an influence on fusion, both qualitative and quantitative fusion assays were performed. For qualitative fusion assays, either MDCK-2 (Fig. 7A) or Vero 76 (Fig. 7B) cells were co-transfected with plasmid DNA encoding for (mutant) CedV G and CedV F. Cells were incubated for 48 h before staining with Giemsa staining solution. As shown in Fig. 7A, syncytium formation is present for CedV G wt as well as for all mutants. However, variation in size and number of the syncytia can be observed for some of the mutants. While mutants e1, e3, and e5 present syncytia comparable in number and size to the parental G, the number of syncytia for e2 and e4 appears reduced. Mutants e6 and e7 only show very few overall syncytia, which additionally seem reduced in size. Syncytium formation in Vero 76 cells (Fig. 7B) presents a slightly different phenotype: syncytia generally seem more prominent and larger than in MDCK-2 cells. However, the comparative results between parental G and mutants are similar to those in MDCK-2 cells. Mutants e1, e3, and e5 seem comparable to the parental G in number and size, while e2, e4, e6, and e7 appear to be reduced in number. The mutants e6 and e7 additionally seem reduced in size. For quantification, we then counted the number of syncytia and the number of cell nuclei per syncytium at 20× magnification in five representative vision fields, and values from five replicates were averaged. The findings for MDCK-2 cells (Fig. 7C) are similar to those found in Vero 76 cells (Fig. 7D), with a moderate decrease in syncytium formation for mutants e2 and e4, while syncytium numbers for e6 and e7 are strongly reduced (no significance, adjusted *P* values ranging from 0.0632 to >1). Mutant e5 additionally shows minor reductions in syncytium formation in Vero 76 cells. Quantification of syncytium size only shows a decrease for e6 and e7 (no significance, adjusted *P* values ranging from 0.0632 to >1). For a more precise evaluation of fusion activity, a quantitative, luciferase reporter gene-based fusion assay was performed in Vero 76 cells after 48 h of incubation (Fig. 8). While mutants e1 and e3 are slightly hyperfusogenic (no significance, adjusted *P* values > 1), mutants e2, e4, and e5 are comparable to the parental CedV G. A strong, though non-significant decrease (adjusted *P* values > 1) of fusion activity was confirmed for mutants e6 and e7. In summary, and particularly when considering the semi-quantitative data (Fig. 7C and D), mutants lacking the di-tyrosine motif (Y_47_Y_48_), either alone or in combination with a missing di-leucine (L_59_L_60_) and/or a YXXN motif (Y_9_XXN), seem to exhibit slightly impaired biological function, although these effects are not statistically significant. Single disruption of the di-leucine or YXXN motif does not appear to impact the biological activity—in this case, mediating fusion—of the respective G proteins.

**Fig 9:**
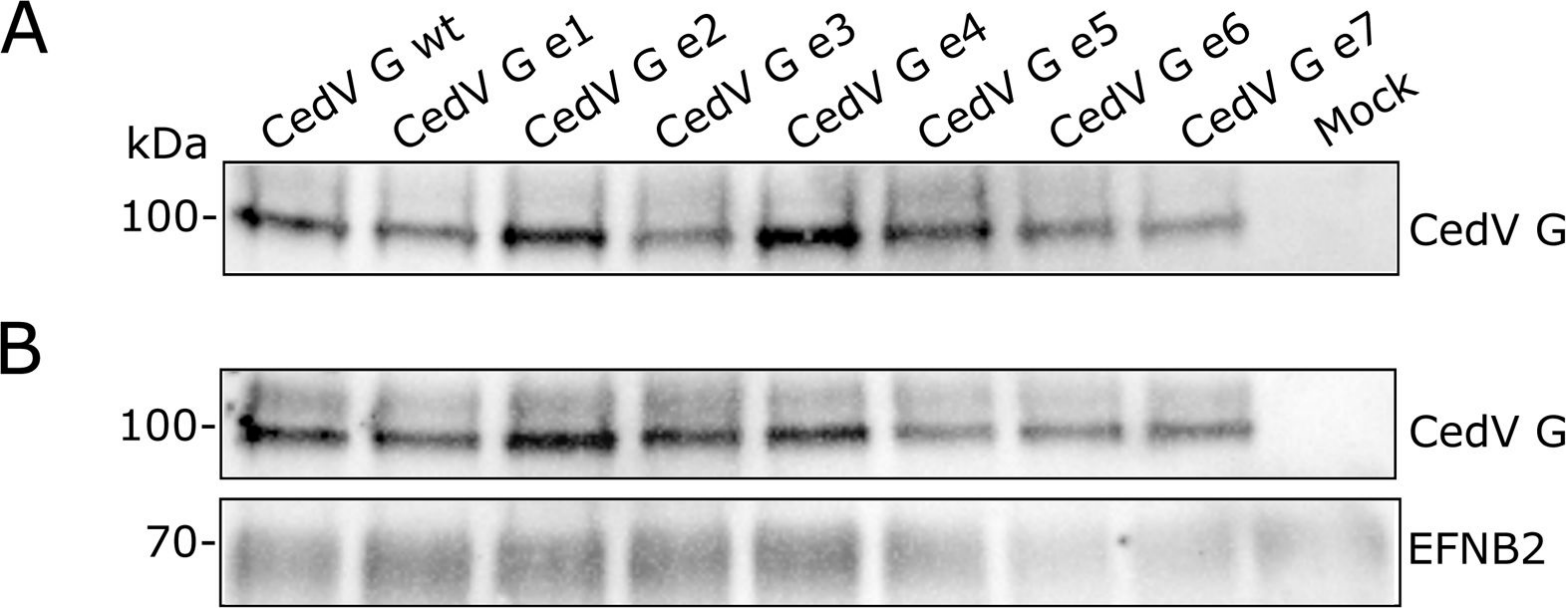
Binding of CedV G proteins to EFNB2. (**A**) Overall CedV G protein expression was determined by acetone precipitation of cell lysates and visualized in Western Blot analysis with anti-HA tag-specific primary and HRP-labeled secondary antibodies. (**B**) CedV G proteins expressed in MDCK-2 cells were immunoprecipitated with soluble human EFNB2-Fc-Tag (1:100) and visualized in Western Blot analysis with anti-HA tag-specific primary (1:1,000), anti-EFNB2 (1:500) and HRP-labeled secondary antibodies. *n* = 3.

**Fig 10:**
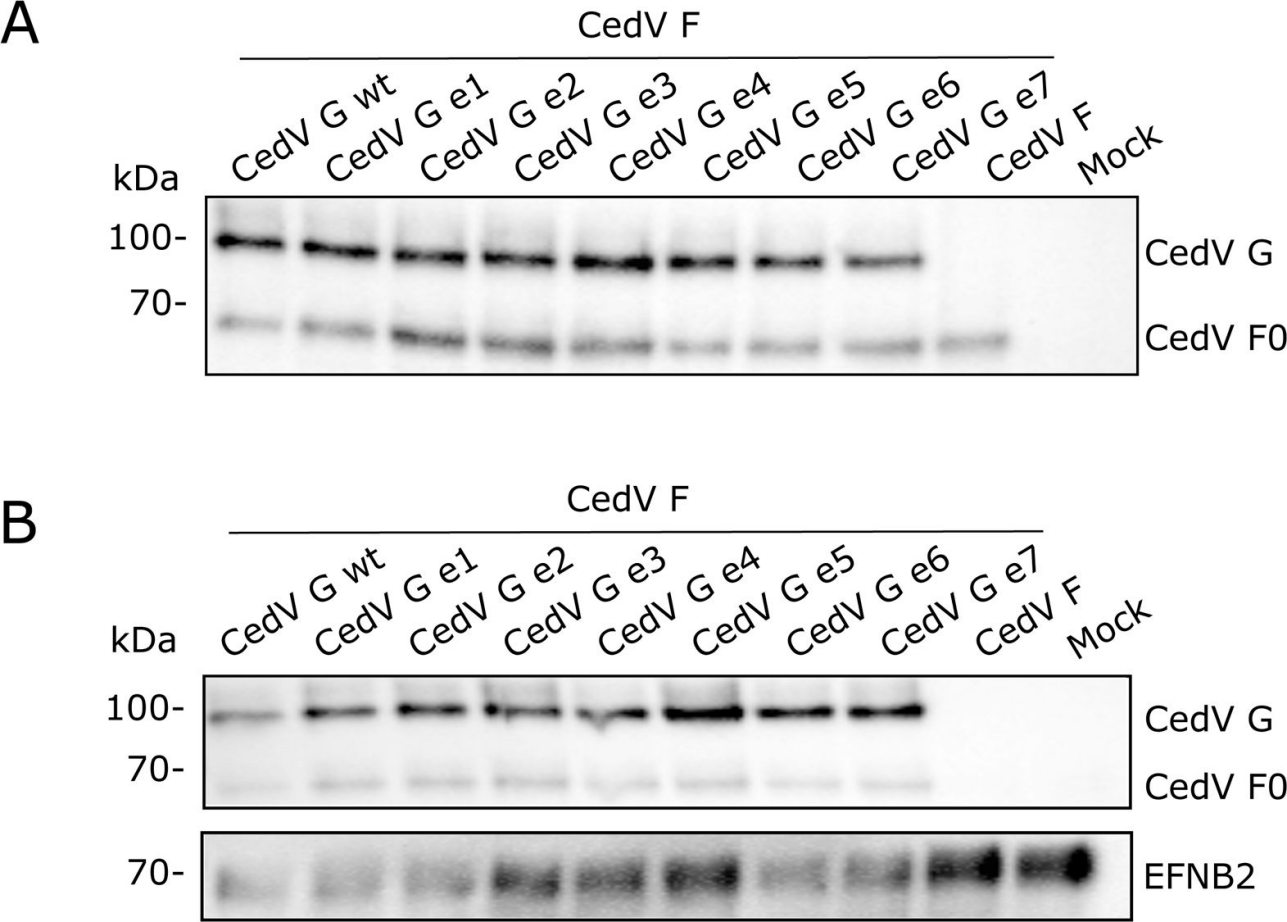
Binding of CedV G co-expressed with CedV F proteins to EFNB2. (**A**) Overall CedV G and F protein expression was determined by acetone precipitation of cell lysates and visualized in Western Blot analysis with anti-HA tag-specific primary and HRP-labeled secondary antibodies. (**B**) CedV G proteins expressed in MDCK-2 cells were immunoprecipitated with soluble human EFNB2-Fc-Tag (1:100). Both G and F proteins were visualized in Western blot analysis with anti-HA tag-specific primary (1:1,000) or EFNB2 with an anti-EFNB2 (1:500) and HRP-labeled secondary antibodies. *n* = 3.

The prerequisite for cell entry and cell-to-cell fusion is binding of the G protein to the host cell receptor, followed by triggering conformational changes in the fusion protein. We demonstrated in Fig. 7 and 8 small differences in fusion formation between the G mutants, but also showed in Fig. 6 that transport of F to the cell surface and F processing seemed to be unaffected. We thus wanted to assess whether alterations in the cytoplasmic domain of CedV G influence receptor binding, in this case binding to EFNB2, which has been described as one of the main utilized receptors for CedV (8). For this, CedV G (mutant) proteins were transiently expressed in MDCK-2 cells, and after cell lysis, G proteins were immunoprecipitated with soluble human EFNB2 and subjected to SDS-PAGE under reducing conditions (Fig. 9). CedV G proteins and EFNB2 were stained with an anti-HA tag-specific primary and an anti-EFNB2 antibody, respectively, and detected with HRP-labeled secondary antibodies. Acetone precipitates demonstrate expression of G proteins in all samples (Fig. 9A). In Fig. 9B, immunoprecipitated proteins of the expected size of 100 kDa are detected for all mutants, which show their capacity of binding to EFNB2 in a similar way as the parental CedV G. To assess whether the co-expression of CedV F has an impact on the receptor binding capability of CedV G mutants, an immunoprecipitation of CedV G from MDCK-2 cells co-expressing F and G with EFNB2 was performed as described above. As demonstrated by the acetone precipitates shown in Fig. 10A, CedV G or G mutants and CedV F are expressed in all samples. As depicted in Fig. 10B, bands of a size of 100 kDa were detected for parental and all mutant CedV G proteins, independent of their co-expression with CedV F, indicating that EFNB2 binding of G mutants was not affected. In addition, F0 bands of a size of approximately 66 kDa were also detected for all samples, hinting at co-precipitation of F and, thus, indirectly suggesting a functional interaction of CedV F with all CedV G mutants. Therefore, a defect in receptor binding or impaired interaction of the CedV G mutants with CedV F does not explain the minor differences in fusion activity observed for some of the mutants.

## DISCUSSION

Endocytosis plays a critical role in the internalization of viral proteins from the cell surface, influencing their activation and biological function. While the role of endocytosis in the maturation and activation of fusion (F) proteins of henipaviruses, such as HeV and NiV, is well established (11–13, 15), the contribution of endocytosis to the function of the attachment (G) glycoproteins remains less clear. Table 1 summarizes an overview of findings from published studies of the endocytosis motifs in the cytoplasmic domains of HeV, NiV, and CedV glycoproteins, compared to the results from this study. Classical endocytosis motifs in the cytoplasmic domains of viral proteins, including YXXΦ and di-leucine motifs, serve as important signals for endocytosis (22) and have been extensively characterized in F proteins, for which clathrin-mediated uptake and subsequent proteolytic activation are essential steps (13, 14, 16, 17).

**TABLE 1:** Comparative assessment of the role of endocytosis motifs in the cytoplasmic domains of henipavirus glycoproteins^*a*^

	CedV G	CedV F	NiV G	NiV F	HeV F
Putative endocytosis motifs	Y_9_XXN; Y_47_Y_48_; L_59_L_60_	Y_522_XXN; Y_524_XXF; Y_533_Y_534_; Y_553_Y_554_	Y_28_Y_29;_ L_41_L_42_	Y_525_XXL; Y_542_Y_543_	Y_525_XXL, Y_542_Y_543_
Protein internalization	Yes, endocytosis? Not quantified	Yes, endocytosis	Yes, membrane turnover	Yes, endocytosis	Yes, endocytosis
Effect of motifs on endocytosis	Not observed	Endocytosis rates reduced when any of the motifs disrupted	Not observed	Endocytosis hindered by disrupted Y_525_XXL; rates reduced when Y_542_Y_543_ disrupted	Endocytosis rate reduced when Y_525_XXL disrupted
Effect on surface levels	Not observed	Marginally increased when Y_524_XXF, Y_533_Y_534_, or Y_553_Y_554_ disrupted	Not observed	Increased levels correlate with decreased internalization rates	Increased when Y_525_XXL disrupted
Effect on fusion activity	Slightly reduced when Y_47_Y_48_ disrupted, accumulative effect by disruption of all motifs	Fusogenicity increased by disrupted Y_524_XXF; reduced when all motifs disrupted	None	Syncytium size reduced when Y_525_XXL disrupted	Increased when Y_525_XXL disrupted
Effect on sorting	n.a.	n.a.	Basolateral sorting affected in epithelial cells by Y_28_Y_29_	Basolateral sorting affected in epithelial and endothelial cells by Y_525_XXL	n.a.
Effect on receptor (EFNB2) binding	Not observed	n.a.	n.a.	n.a.	n.a.

^*a*^ HeV G was not included in this comparison, since no endocytosis motifs are present in its cytoplasmic domain. The data included in this table is based on findings from Fischer et al. (24), Vogt et al. (11), Meulendyke et al. (12), Erbar et al. (28), and Weise et al. (29). n.a., not assessed.

However, the role of such motifs in the CedV G protein, which contains several canonical motifs (YXXN, di-tyrosine, and di-leucine), has not been elucidated. Our study aimed to determine whether these motifs have an impact on endocytosis and, subsequently, the biological activity of CedV G, including receptor binding and triggering of the F protein for fusion. Interestingly, although CedV G undergoes endocytosis, this process appears to occur independently of the classical motifs analyzed in this study. In contrast, these motifs do not affect CedV G expression or cell surface transport, nor the transport or proteolytic activation of CedV F. Notably, mutation of a di-tyrosine motif (Y_47_Y_48_) in CedV G was observed to slightly reduce fusion activity, suggesting a certain role of this motif beyond classical endocytosis signaling.

Even though CedV G undergoes endocytosis, none of the potential endocytosis motifs seems to impair the uptake from cell surface. All evaluated mutants, independently of whether they contained single or combined mutations, were taken up into the cell within a period of 30 min. This differs from other viral glycoproteins, where similar motifs have been identified. Especially, tyrosine-based motifs have often been found to initiate and regulate endocytosis. The YXXΦ motif was identified as the major endocytosis signal for CedV F (Y_524_XXF) and NiV F (Y_525_XXL), although the di-tyrosine motifs (Y_533_Y_534_ and Y_553_Y_554_ for CedV F, and Y_542_Y_543_ for NiV F) also appear to influence uptake rates, albeit to a lesser extent. In both cases, the triple mutants, which lacked all tyrosine motifs present in the cytoplasmic domain, showed an almost complete loss of endocytic uptake (11, 24). However, for CedV F, the individual mutation of the tyrosine motif Y_522_XXN and the combined mutations of Y_522_XXN and Y_524_XXF resulted in reduced internalization rates, with endocytosis measured after 15 to 30 min in a quantitative endocytosis assay, whereas the parental protein was already detectable in the cell after 5 min (24). The degenerate YXXN motif differs from the classical canonical YXXΦ motif, as this motif displays an asparagine at its fourth position instead of an amino acid with a bulky hydrophobic side chain, which is normally characteristic for these motifs. Interestingly, the disruption of the degenerate tyrosine motif Y_12_XXN in MEV H equally led to reduced internalization rates of <1%/min, whereas the parental H protein was taken up at a rate of 2.5%/min, suggesting this motif also as an important endocytosis signal, aside from the classical canonical motifs (26). In contrast to these findings, the internalization of NiV G has been described as independent of the di-tyrosine and di-leucine motifs present in its cytoplasmic domain (11). Consistent with our results, the study demonstrated that disruption of these motifs in the G protein did not prevent its uptake into cells. Moreover, the uptake of the NiV G protein occurred at a rate of 1%/min, which is in line with the general membrane turnover. Whether CedV G is likewise internalized as part of general membrane turnover, however, remains to be determined (11). Nonetheless, the di-tyrosine motif has been demonstrated to play a role in the correct basolateral sorting of the protein in epithelial cells, while the di-leucine motif has not shown this effect (28, 29). Whether this also applies to the motifs in the cytoplasmic domain of CedV G needs to be clarified in further studies with polarized cells.

Signal-mediated endocytosis does not only contribute to the maturation and activation of viral proteins but is also thought to function as a regulatory mechanism to avoid excessive expression of glycoproteins on the cell surface. Viruses often tightly control and minimize the expression of surface glycoproteins to lessen cytotoxic effects and evade immune responses triggered by the complement system or antibody-mediated mechanisms. To analyze whether the motifs in this study have an influence on cell surface expression, cell surface biotinylation assays and imaging-based quantification from cell surface immunofluorescence were performed. Our findings only show marginal differences in the expression of proteins on the cell surface, which can most likely be explained through methodological variation rather than through real differences in expression levels. For CedV F, the mutant with a disrupted YXXN motif did not lead to any changes in cell surface expression, while single disruptions of the YXXF and di-tyrosine motifs, as well as the combined disruption of YXXN and YXXF, led to marginal increases. However, a marked increase of surface expression was observed for the mutant lacking both di-tyrosine motifs and for the mutant in which all motifs had been disrupted. This could be explained by an accumulation on the surface due to reduced internalization or by an impairment in endosomal trafficking and recycling (24). Similar observations were made for NiV F and HeV F mutants lacking the YXXΦ motif (11, 12).

Although no alterations in the internalization nor in cell surface expressions could be observed, decreases in biological activity, assessed through syncytium formation, could be detected for some mutants. Interestingly, all mutants showing impaired fusion activity are lacking the di-tyrosine motif, and the mutant e6, with all motifs disrupted, displays the strongest decrease in fusion activity. A multitude of factors may provide a rationale for the observed results. First, for the successful entry into host cells, binding to the host cell receptor is necessary. In the case of CedV, the utilized receptors are EFNB1, -B2, -A2, and -A5, whereas the strongest affinity has been described for EFNB2 (8). Our data show that all mutants are capable of binding to EFNB2, both when expressed individually and when co-expressed with CedV F. Second, the co-expression on the cell surface and the interaction of F and G (25) are another prerequisites for physiological functionality. Cell surface biotinylation shows that F protein surface expression is unaffected by the co-expressed G mutant. Cleavage of F into F0 and F1 is also comparable after co-expression with G mutants or parental CedV G, and surface levels of CedV G mutants also remain unchanged when co-expressed with CedV F. This suggests that the disruption of the motifs in CedV G does not influence the transport and proteolytic activation of CedV F. These results align with findings for the Hendra virus glycoproteins, which show that the trafficking of HeV F and G proteins to the cell surface occurs independently (30, 31). In addition, the interaction on the plasma membrane of both glycoproteins seems to play a role in the biological functionality as described for NiV G and F (32). As illustrated by the example of NiV, specific signals within the cytoplasmic domain can have an impact on this interaction. A mutation of a KKR motif in NiV F resulted in a diminished detection of the protein when co-immunoprecipitated through G (33). The avidity of NiV F and G additionally inversely correlates with the fusogenicity. The weaker the interaction of both proteins, the easier the dissociation from the receptor, which leads to increased fusion activities (34, 35). Similar findings were described for measles virus: tagged forms of MeV H (flag- or HA-tagged) presented a reduced interaction with MeV F in infected cells compared to untagged MeV H. Interestingly, this weakened interaction also resulted in increased viral fusogenicity (36). In contrast, fusion assays of this study did not reveal a marked increase of fusion activities for any of the mutants. These findings are consistent with the results from the co-expressed CedV F and G mutants precipitated with EFNB2, which demonstrated that an interaction of CedV F occurs with all of the CedV G mutants. However, the data do not allow interpretation regarding the avidity of the interaction. It is clear that further studies will be required to clarify how the disruption of the motifs in the cytoplasmic tail of CedV G contributes to a decrease in biological activity.

In this study, we demonstrated that the CedV G protein is taken up into cells during CedV infection and following transient expression of CedV G alone. Furthermore, the cytoplasmic domain of CedV G contains three putative endocytosis motifs, consistent with the observations for other henipavirus glycoproteins. Using multiple transfection-based assays, we showed that mutation of these motifs does not affect CedV G expression, surface transport, EFNB2 receptor binding, or interaction with CedV F. However, a mechanistic explanation for the moderately reduced fusion activity observed for some mutants, particularly those involving disruption of the Y_47_Y_48_ motif, could not be established within the scope of this study. Future studies using F/G-pseudotyped viruses or recombinant viruses will be necessary to assess how these properties are modulated during viral infection. Previous studies on other henipavirus glycoproteins have indicated that disruption of individual endocytosis motifs does not necessarily abolish internalization but can instead influence the rates of endocytosis (11, 12, 24). Whether the motifs identified in CedV G have a similar regulatory effect on endocytosis rates requires quantitative endocytosis assays and therefore remains to be addressed in future studies. Additionally, classical endocytosis motifs can play a role beyond mediating internalization. For NiV G, which is taken up via membrane turnover, a di-tyrosine motif was found to be critical for the correct basolateral sorting (11). Whether a comparable role also applies for CedV G will need to be investigated using polarized cell systems. Finally, the cytoplasmic domains of henipavirus glycoproteins F and G are known to mediate interactions not only with each other but also with the matrix protein M, which is a key driver of viral budding (37). Thus, it is conceivable that altered interactions between CedV G mutants and the M protein might contribute to a reduced biological activity observed for certain mutants. This possibility could be addressed in future studies using virus-like particle systems or recombinant viruses.

### Conclusion

In summary, our findings show that CedV G is taken up into the cell. However, it appears that endocytosis occurs independently of signals and, thus, regardless of specific endocytosis motifs in a manner similar to that observed for NiV G. Nevertheless, the di-tyrosine signal Y_47_Y_48_ seems to have an impact on the biological activity of the glycoprotein G. How this motif interacts with other viral proteins and, thus, influences the cell fusion needs to be investigated in the future. Furthermore, the role of the motifs for protein sorting and trafficking to the apical or basolateral membrane needs to be investigated.

## MATERIALS AND METHODS

### Cell lines, transfection, and infection

Vero 76 and Madin-Darby Canine Kidney 2 (MDCK-2) cells (Collection of Cell Lines in Veterinary Medicine, Friedrich-Loeffler-Institut, FLI; CCLV-RIE 0228 and 1061, respectively) were maintained in Dulbecco’s modified Eagle’s medium (DMEM) supplemented with fetal calf serum (10% and 5%, respectively) and incubated at 37°C. Vero 76 and MDCK-2 cells were reverse-transfected by using the Lipofectamine 3000 reagent (Invitrogen) following the manufacturer’s protocol.

MDCK-2 cells (1.2 × 10^5^ cells/well) were infected with recombinant CedV wild-type (rCedV) based on the CedV isolate CG1a genome sequence (GenBank accession no. NC_025351.1) (kindly provided by Stefan Finke, Friedrich-Loeffler-Institut [38]) at a multiplicity of infection (MOI) of 0.01 in DMEM. One hour post-infection, medium was changed to DMEM supplemented with 5% FCS. Work with rCedV was approved by the State Office for Health and Social Affairs Mecklenburg-Western Pomerania under the references LAGuS3021_04/11.6.19 and LAGuS3021_04/11.6.19 (E1) and is performed under BSL2-conditions.

### Plasmids and site-directed mutagenesis

CedV F and G (GenBank accession no. NC_025351.1) open reading frames (ORF) were synthesized by GeneArt (Thermo Fisher Scientific Inc.) and subcloned into the pCAGGS expression vector. The CedV G ORF was codon-optimized for expression in human cells and HA-tagged at the C-terminus and will be referred to as wild-type (wt) in the manuscript. Site-directed mutagenesis was performed on selected tyrosine and leucine residues, substituting them with alanine within the cytoplasmic domain of the CedV G protein, using the QuikChange Lightning Site-Directed Mutagenesis Kit (Agilent). Primers were designed according to the manufacturer’s instructions. All mutants were confirmed by Sanger sequencing.

### Antibody uptake assay

MDCK-2 cells (1.5 × 10^5^ cells/well) were transfected with plasmid DNA (1 µg/well) encoding for CedV G or CedV G mutant protein genes. At 24 h p.t., cells were washed with phosphate-buffered saline supplemented with CaCl_2_ and MgCl_2_ (PBS++) and incubated for 1 h at 4°C without prior fixation with a polyclonal anti-HA tag antibody (H6908; 1:500 dilution in 0.35% BSA in PBS++; Sigma-Aldrich) or with polyclonal anti-CedV G rabbit serum to detect untagged protein (1:2,000 dilution in 0.35% BSA in PBS++). After another washing step, cells were either left at 4°C or shifted to 37°C for 30 min with prewarmed medium to allow endocytosis to occur. Surface-bound primary antibodies were then stained with an Alexa Fluor (AF) 488 goat-anti-rabbit antibody in surplus (1:50 in 0.35% BSA in PBS++, Life Technologies) for 90 min at 4°C. Finally, cells were fixed and permeabilized using methanol-acetone (1:1) to allow staining of internalized primary antibodies with an AF 568 goat-anti-rabbit antibody (1:500 in 0.35% BSA in PBS++, Life Technologies) for 35 min at 4°C. Representative images were acquired using a Nikon Eclipse Ti-S microscope at 60× magnification and processed with ImageJ software (version 1.54p).

The antibody uptake assay with rCedV-infected cells was performed as described above, with the only difference being that anti-CedV G-specific monoclonal primary antibodies produced in-house and goat-anti-mouse secondary antibodies were used.

For imaging-based quantification of G cell surface expression, the control cells that were left at 4°C are used. Representative images of five fields of view for each sample were captured with a Nikon Eclipse Ti-S at 20× magnification and analyzed using the Fiji ImageJ software (version 1.54f). Surface pixels and integrated densities of all objects (cells) with sizes of 200 to 327,680 voxels and a threshold of 30 were determined. The mean densities of every cell were calculated, and the average density of the overall cell surface fluorescence of all five fields of view was defined. The mean densities of parental CedV G-expressing cells served as a reference and were set to 1 for better comparison of the expression levels of the CedV G mutants on the cell surface.

### Indirect immunofluorescence assay

MDCK-2 cells (1.5 × 10^5^ cells/well) were transfected with plasmid DNA (1 µg/well) encoding for CedV G or CedV G mutants. At 24 h p.t., cells were washed with PBS++ and fixed with ice-cold methanol-acetone. Staining was performed with the H6908 primary anti-HA tag antibody (1:500 in 0.35% BSA in PBS++) and a secondary AF 568 goat-anti-rabbit antibody (1:500 in 0.35% BSA in PBS++, 1 h at 4°C each). Cell nuclei were stained using 4',6-diamidino-2-phenylindole dihydrochloride (DAPI, 1:20,000 in PBS, 10 min at 37°C; Carl Roth). Representative images were acquired with a Nikon Eclipse Ti-S microscope at 60× magnification and edited using the ImageJ software (version 1.54p).

### Western blot analysis

MDCK-2 cells (1.5 × 10^6^ cells/well) were transfected with plasmid DNA (5 µg/well) encoding for CedV G or mutant CedV G. At 30 h p.t. cells were washed with PBS and lysed with SDS-sample buffer containing Tris/HCl (pH 6.8), sodium dodecyl sulfate (SDS), glycerin, bromphenol blue, and 2-mercaptoethanol. Proteins were separated on a 10% SDS-gel under reducing conditions and transferred onto a nitrocellulose membrane. Proteins were then incubated with an anti-HA tag antibody (H6908, 1:500 in PBS-Tween 0.05% [PBS-T]) overnight at 4°C. For detection, a goat-anti-rabbit HRP-conjugated secondary antibody (1:5,000 in PBS-T, 1 h at RT; Life Technologies) was used. GAPDH was used as an internal loading control (anti-GAPDH antibody, 1:5,000 in PBS-T, overnight; Sigma-Aldrich), followed by incubation with a goat-anti-rabbit HRP-conjugated secondary antibody (1:5,000 in PBS-T, 1 h). Proteins were visualized with chemiluminescent substrate (Clarity Western ECL substrate, Bio-Rad) using the Bio-Rad Molecular Imager ChemiDoc XRS+ in combination with Image Lab software (Bio-Rad, Version 6.0.1).

### Cell surface biotinylation and Western blot analysis

MDCK-2 cells (1.5 × 10^6^ cells/well) were either transfected with plasmid DNA encoding (mutant) CedV G (5 µg/well) or co-transfected with both CedV F and (mutant) CedV G (2.5 µg/well each for co-cell surface biotinylation). At 30 h p.t., cells were washed with PBS++ and incubated with 1.2 mg EZ-Link Sulfo-NHS-LC-Biotin (ThermoFisher Scientific) for 30 min at 4°C. After cell lysis, the proteins were immunoprecipitated overnight with NeutrAvidin-Beads (ThermoFisher Scientific), then separated on a 10% SDS-gel under reducing conditions, and transferred onto a nitrocellulose membrane. Biotinylated CedV G proteins were incubated with the H6908 anti-HA tag antibody in a dilution of 1:500 in PSB-T overnight at 4°C. An anti-rabbit HRP-conjugated secondary antibody (1:5,000 in PBS-T 0, 1 h at RT) was used for detection. Sodium/potassium-transporting ATPase subunit beta 1 (ATP1B1) served as a loading control and was detected with an anti-ATP1B1 antibody (1:5,000 in PBS-T, Invitrogen) followed by anti-mouse HRP-conjugated secondary antibody labeling. Proteins were visualized with chemiluminescent substrate (Clarity Western ECL substrate, Bio-Rad) using the Bio-Rad Molecular Imager ChemiDoc XRS+ in combination with Image Lab software (Bio-Rad, Version 6.0.1).

### Qualitative fusion assay

Vero 76 cells (2 × 10^5^ cells/well) or MDCK-2 cells (1.3 × 10^5^ cells/well) were co-transfected with plasmid DNA encoding for CedV F (0.25 µg/well) and either CedV G or mutant CedV G (0.75 µg/well). Cells were fixed with ethanol at 48 h p.t. and stained with Giemsa solution (1:10 dilution, Carl Roth). Representative images were documented (20× magnification) using a Nikon Eclipse TS100 microscope. For each mutant, the number of syncytia and the number of cell nuclei per syncytium were counted in five representative vision fields of each independent biological replicate, and values of five replicates were averaged. One syncytium was defined as consisting of at least five nuclei. The parental CedV G served as a reference and was set to 1 for comparison of the mutated CedV G proteins.

### Luciferase reporter gene-based fusion assay

The quantitative fusion assay in Vero 76 cells was performed as described previously (24) with slight modifications. Briefly, Vero 76 cells (1.5 × 10^5^ cells/well) were co-transfected with plasmids encoding for pCite Renilla (200 ng/well), CedV F (125 ng/well), and CedV G or mutant CedV G (375 ng/well) in 24-well plates. At the same time, 5 × 10^6^ Vero 76 cells were transfected with plasmid DNA encoding for pCAGGS T7 polymerase (10 µg) in a petri dish. After an incubation period of 48 h, T7-expressing cells were detached using 5 mM EDTA, and 2 × 10^5^ cells were added onto the CedV F and G co-transfected cells to allow fusion of the cells. After a 3-h incubation period, cells were washed with PBS and incubated with 200 µL 1× Lysis-Juice (pjk GmbH) for 15 min at RT. After that, 40 µL of the cell lysates were mixed with the same volume of Renilla GLOW-Juice (pjk GmbH), and luciferase activity was immediately measured using a Berthold Technologies Bioanalytics Centro LB 963 Microplate Luminometer. Cells co-transfected with pCite Renilla only or pCite Renilla and CedV G served as background control. CedV G wt and CedV F co-transfected cells served as the reference, and the measured luciferase activity was set to 1 for comparison with the G mutants.

### Immunoprecipitation with recombinant EFNB2 and Western blot analysis

MDCK-2 cells (1.5 × 10^6^ cells/well) were either transfected with plasmid DNA encoding for CedV G or mutant CedV G, or co-transfected with plasmid encoding for HA-tagged CedV F (CedV F) and CedV G wt or mutant CedV G (co-IP of CedV F and CedV G wt or G mutants with EFNB2). At 24 h p.t., cells were harvested in 1 mL PBS and centrifuged for 4 min at 2,000 rpm at 4°C. The supernatant was discarded, and pellets were lysed in 1 mL lysis buffer (containing 5 M NaCl, 1 M Tris HCl pH 7.4, 10% NP-40, H2O, and Protease Inhibitor cOmplete [Roche]) under rotation for 2 h at 4°C. Dynabeads Protein G (Invitrogen) were incubated for 30 min at room temperature under rotation with recombinant soluble human ephrin-B2-Fc-Tag (EFNB2) (1:100 in PBS-Tween 0.02% [PBS-T 0.02%]; Sino Biological Inc.) and then washed once with PBS-T 0.02% prior to use. 150 µL of the lysates was precipitated with 750 µL of ice-cold acetone and served as a control for total protein expression before immunoprecipitation (IP). The remaining 850 µL of supernatant from cell lysates was used for IP and incubated for 30 min at room temperature under rotation with the bead-antibody solution. The beads were washed three times and resuspended with PBS-T 0.02%, and then eluted in SDS-sample buffer containing 4% 2-mercaptoethanol. Proteins were separated on 10% SDS-PAGE gel under reducing conditions and transferred onto a nitrocellulose membrane. CedV G and G mutants were visualized with the anti-HA.11 Epitope Tag Antibody (1:1,000 in PBS-T, overnight) and an anti-mouse HRP-conjugated secondary antibody (1:5,000 in PBS-T, 1 h at RT). Incubation with an anti-EFNB2 antibody (Ab-330, Sigma) at a 1:500 dilution and a secondary anti-rabbit HRP-conjugated antibody (1:5,000 in PBS-T 0.05%, 1 h at RT) was performed for staining of EFNB2. Visualization was performed as described above.

### Statistical analyses and data visualization

For all explorative statistical analyses, a Mann-Whitney *U* test was performed using GraphPad Prism version 9.5.1 for Windows, GraphPad Software (Boston, Massachusetts USA, https://www.graphpad.com/). *P* values were corrected using the Bonferroni correction for multiple comparisons to determine statistical significance. The data from each condition was set in relation to the respective reference condition, and the obtained relative values from each replicate were averaged. The results were represented in columns with the standard error of the mean (SEM).

## Data Availability

All data supporting the findings of this study are available within the article or its supplemental material. Further details can be obtained from the corresponding author.

## SUPPLEMENTAL MATERIAL

Fig. S1 to S4 jvi.00187-26-s0001.pdf Additional antibody uptake experiments and additional quantification.

ASM does not own the copyrights to Supplemental Material that may be linked to, or accessed through, an article. The authors have granted ASM a non-exclusive, world-wide license to publish the Supplemental Material files. Please contact the corresponding author directly for reuse.
