# Supplementary material for: Relevance of potential endocytosis motifs in Cedar virus glycoprotein G for its biological activity
Source: J Virol. 2026 Apr 20;100(5):e00187-26. doi: 10.1128/jvi.00187-26 (PMC13185632; doi:10.1128/jvi.00187-26)
Supplement: Fig. S1 to S4 — Additional antibody uptake experiments and additional quantification. [file jvi.00187-26-s0001.pdf]

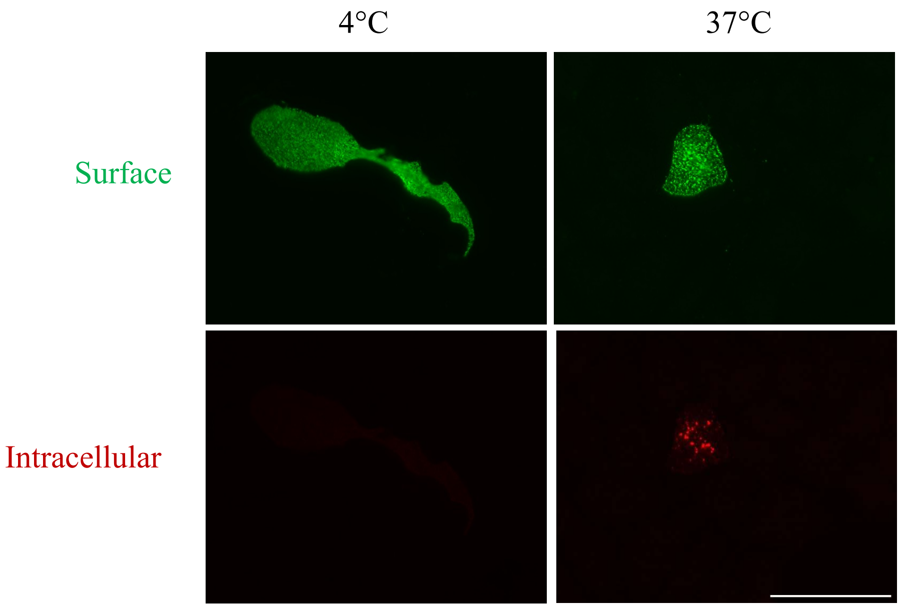

FIG S1 Endocytosis of CedV G protein in transiently transfected MDCK-2 cells. At 24 h p.t., CedV G-expressing cells were incubated without prior fixation with polyclonal anti-CedV G rabbit serum to label G protein on the cell surface. Then, cells were either incubated at 4°C or shifted to 37°C for 30 min to allow endocytosis to occur. Primary antibodies bound to G protein on the cell surface were stained with AF 488-conjugated secondary antibodies. After fixation and permeabilization, internalized primary antibody-CedV G protein complexes were visualized with AF 568-conjugated secondary antibodies. Magnification x60. Scale bar = 50 µm. n=1.

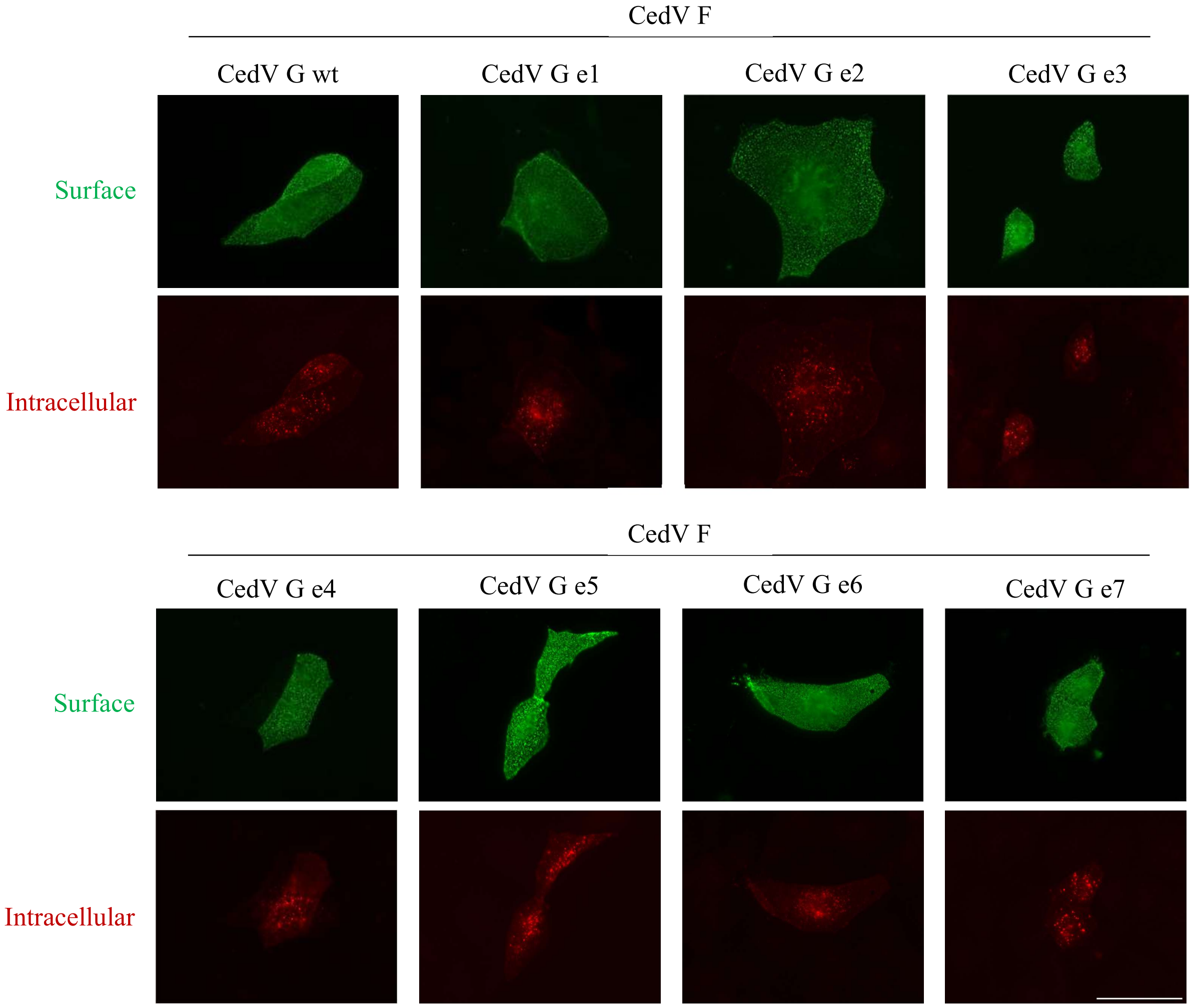

FIG S2 Endocytosis assay in MDCK-2 cells co-expressing CedV G mutants and CedV F. At 24 h p.t., G-expressing cells were incubated without prior fixation with an anti-HA-Tag antibody to label surface-expressed G protein. Then, cells were either incubated at 4°C or shifted to 37°C for 30 min to allow endocytosis to occur. Bound antibodies on the cell surface were stained with AF 488-conjugated secondary antibodies, while endocytosed proteins-antibody complexes were visualized with AF 568-conjugated secondary antibodies after cell fixation and permeabilization. Magnification x60. Scale bar = 50 µm. n=2.

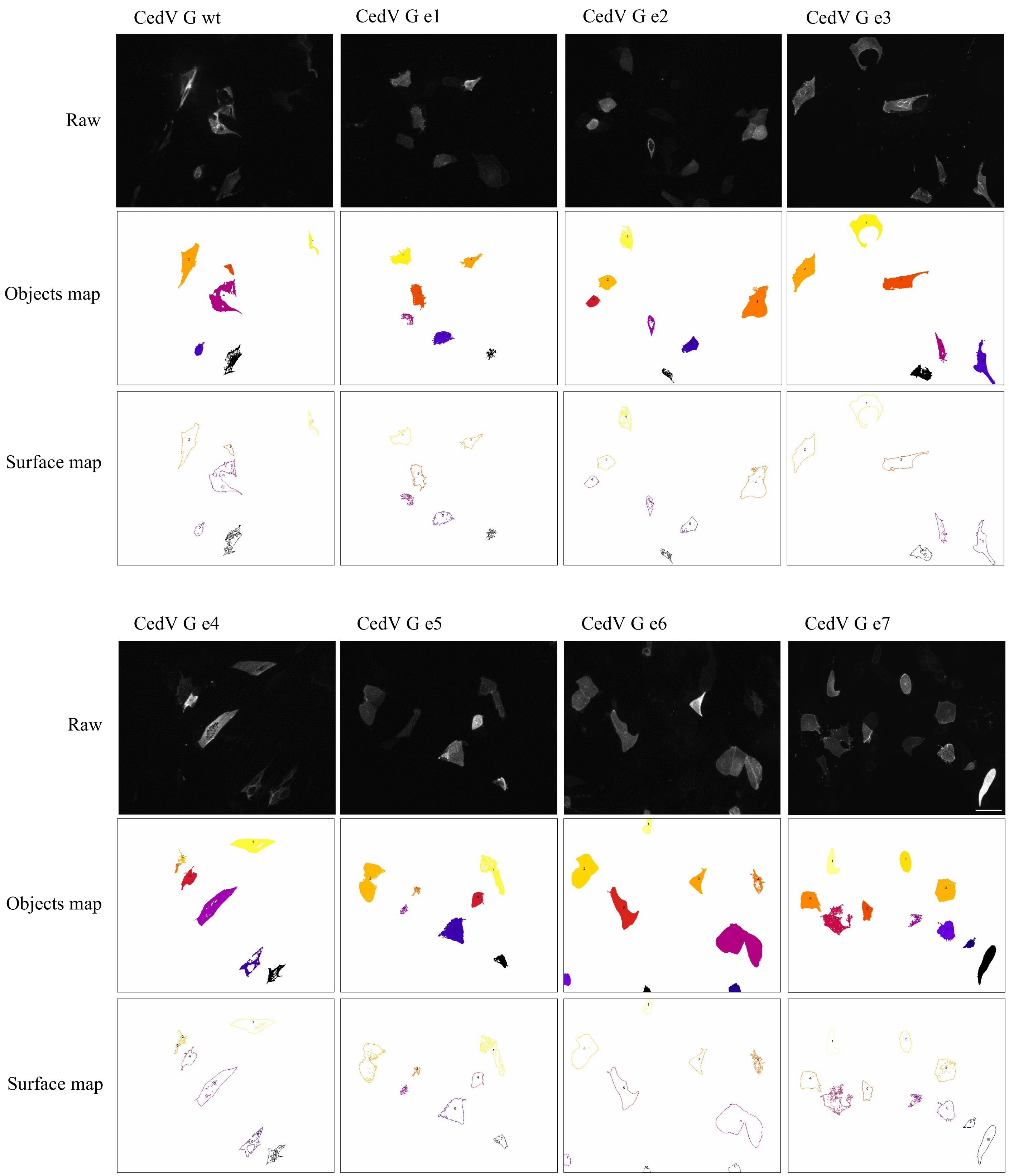

FIG S3 Quantification of cell surface expression by immunofluorescence staining of MDCK-2 cells expressing CedV G (mutant) proteins (4°C controls from endocytosis uptake). Representative images were acquired with a Nikon Eclipse Ti-S microscope at 20x

magnification and analyzed using the Fiji ImageJ software. Surface pixels and integrated densities from 5 fields of view of all objects with a size of 200 to 327680 voxels and a threshold of 30 served to calculate the mean densities shown in Fig. 5C. Scale bar = 50 µm. n=1.

A

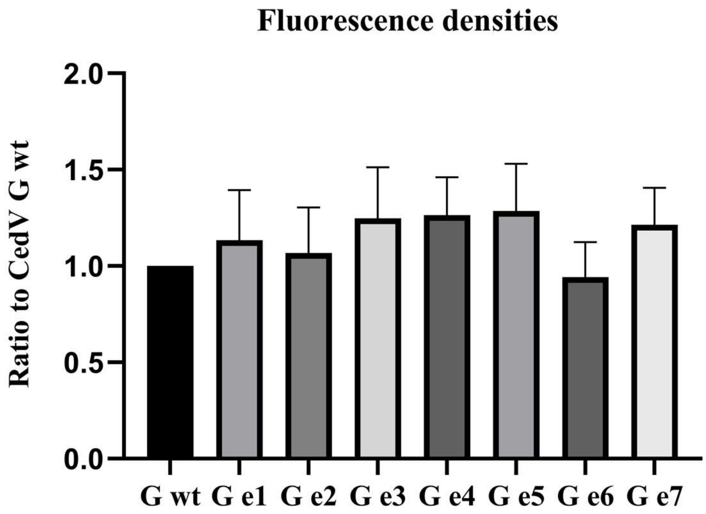
Fluorescence densities
Ratio to CedV G wt
2.0
1.5
1.0
0.5
0.0
G wt
G e1
G e2
G e3
G e4
G e5
G e6
G e7

B

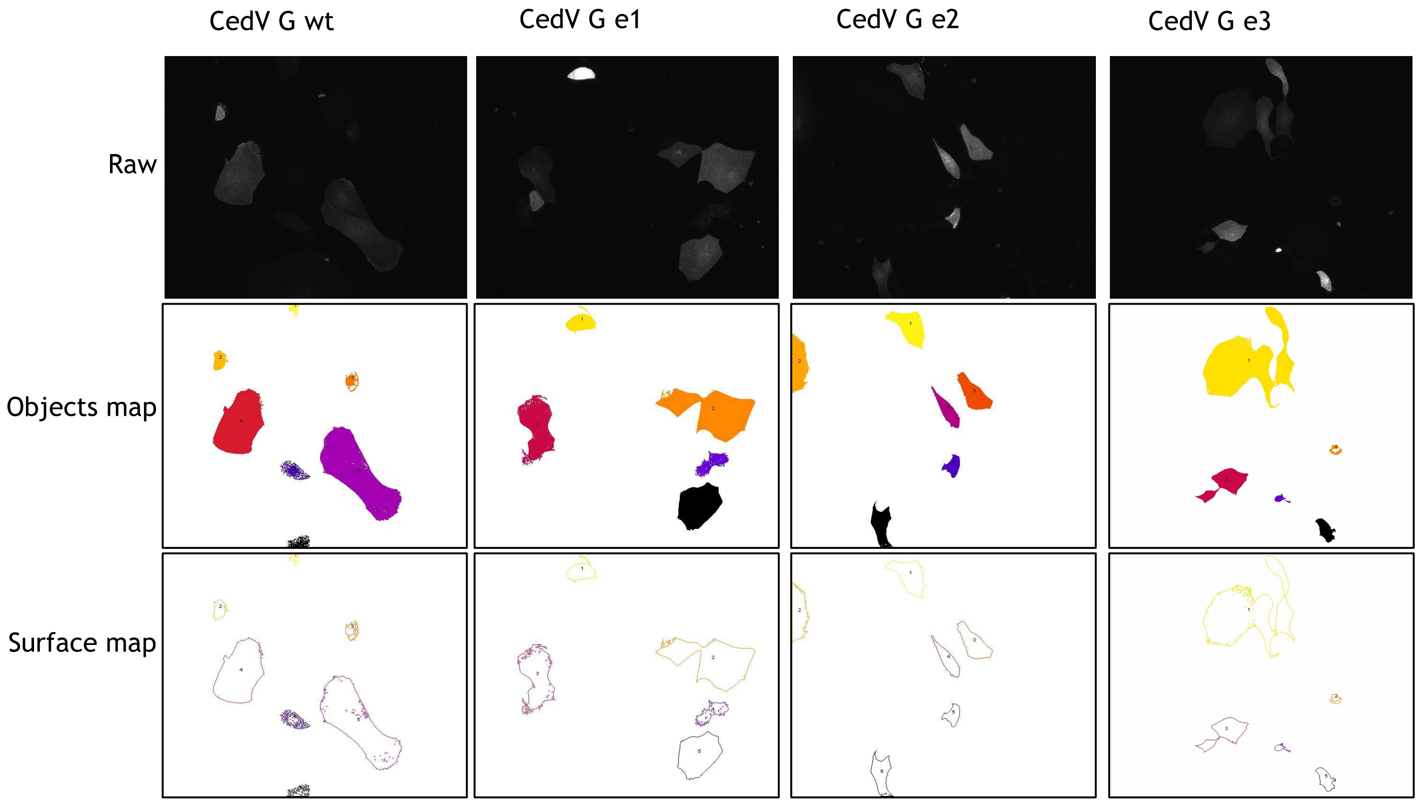
CedV G wt
CedV G e1
CedV G e2
CedV G e3
Raw
Objects map
Surface map

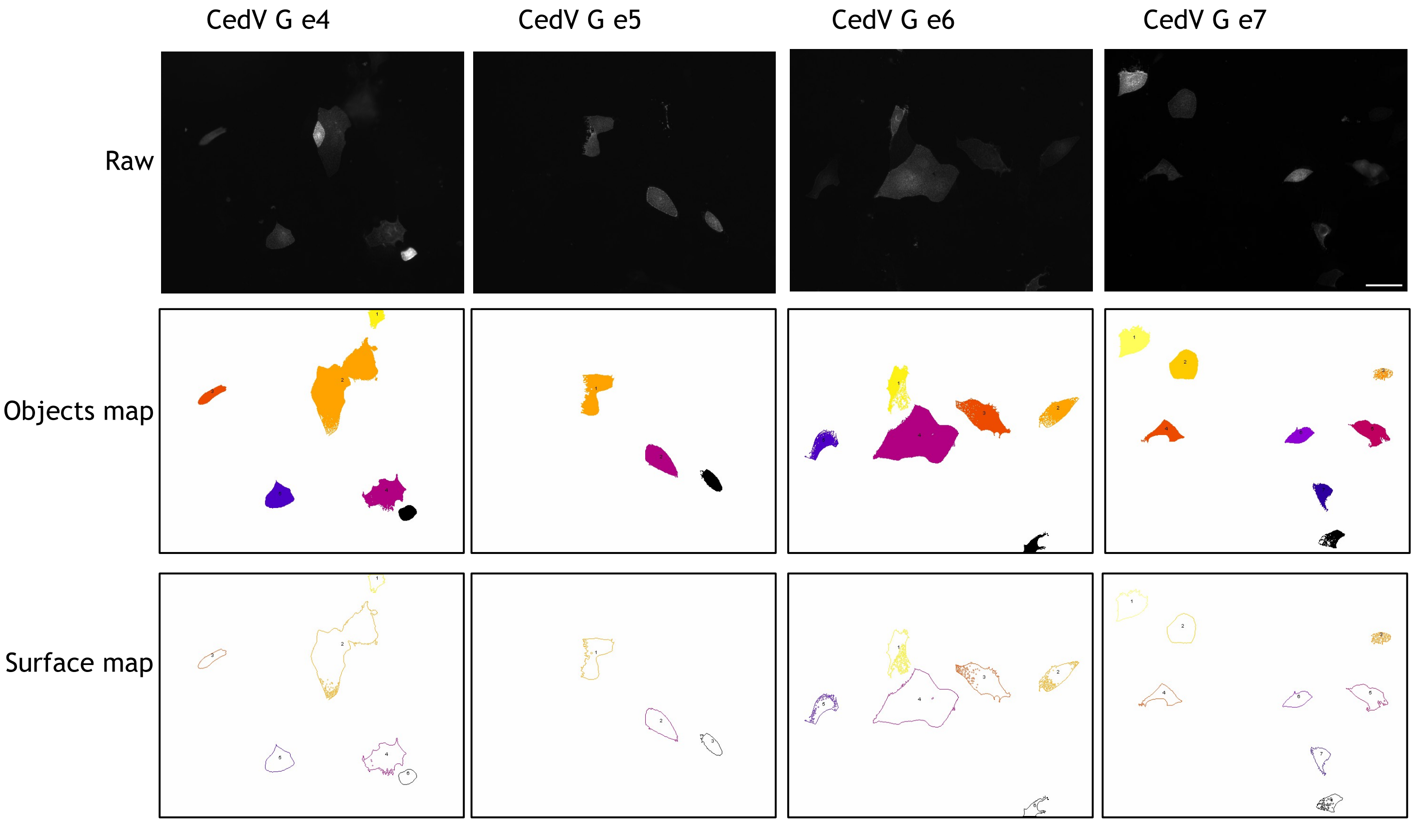

FIG S4 Quantification of cell surface levels of CedV G or mutant G proteins by immunofluorescence staining of MDCK-2 cells co-expressing CedV G (mutant) and CedV F proteins (4°C controls from endocytosis uptake). (A) Surface levels of mutant CedV G proteins set in relation to CedV G wt surface levels. (B) Representative images were acquired with a Nikon Eclipse Ti-S microscope at 20x magnification and analyzed using the Fiji ImageJ software. Surface pixels and integrated densities from five fields of view of all objects with a size of 200 to 327680 voxels and a threshold of 4148 served to calculate the mean densities shown in (A). Scale bar = 50 µm. n=1.
